# Mechanistic characterization of oscillatory patterns in unperturbed tumor growth dynamics: The interplay between cancer cells and components of tumor microenvironment

**DOI:** 10.1371/journal.pcbi.1011507

**Published:** 2023-10-04

**Authors:** Aymara Sancho-Araiz, Zinnia P. Parra-Guillen, Jean Bragard, Sergio Ardanza, Victor Mangas-Sanjuan, Iñaki F. Trocóniz

**Affiliations:** 1 Pharmacometrics & Systems Pharmacology Group, Department of Pharmaceutical Technology and Chemistry, School of Pharmacy and Nutrition, University of Navarra, Pamplona, Spain; 2 IdiSNA, Navarra Institute for Health Research, Pamplona, Spain; 3 Department of Physics and Applied Math. University of Navarra, Pamplona, Spain; 4 Institute of Data Science and Artificial Intelligence, DATAI, University of Navarra, Pamplona, Spain; 5 Department of Pharmacy and Pharmaceutical Technology and Parasitology, Faculty of Pharmacy, University of Valencia, Valencia, Spain; 6 Interuniversity Research Institute for Molecular Recognition and Technological Development, Valencia, Spain; University at Buffalo - The State University of New York, UNITED STATES

## Abstract

Mathematical modeling of unperturbed and perturbed tumor growth dynamics (TGD) in preclinical experiments provides an opportunity to establish translational frameworks. The most commonly used unperturbed tumor growth models (i.e. linear, exponential, Gompertz and Simeoni) describe a monotonic increase and although they capture the mean trend of the data reasonably well, systematic model misspecifications can be identified. This represents an opportunity to investigate possible underlying mechanisms controlling tumor growth dynamics through a mathematical framework. The overall goal of this work is to develop a data-driven semi-mechanistic model describing non-monotonic tumor growth in untreated mice. For this purpose, longitudinal tumor volume profiles from different tumor types and cell lines were pooled together and analyzed using the population approach. After characterizing the oscillatory patterns (oscillator half-periods between 8–11 days) and confirming that they were systematically observed across the different preclinical experiments available (p<10^−9^), a tumor growth model was built including the interplay between resources (i.e. oxygen or nutrients), angiogenesis and cancer cells. The new structure, in addition to improving the model diagnostic compared to the previously used tumor growth models (i.e. AIC reduction of 71.48 and absence of autocorrelation in the residuals (p>0.05)), allows the evaluation of the different oncologic treatments in a mechanistic way. Drug effects can potentially, be included in relevant processes taking place during tumor growth. In brief, the new model, in addition to describing non-monotonic tumor growth and the interaction between biological factors of the tumor microenvironment, can be used to explore different drug scenarios in monotherapy or combination during preclinical drug development.

## Author summary

Mathematical models for tumor growth kinetics have been widely used for several decades, including among others the exponential, the Gompertz and the Simeoni model. However, as the knowledge of the multiple processes taking place during tumor microenvironment increases, models including plausible mechanisms are becoming increasingly important. In this work, we highlight the oscillatory dynamics observed in the tumor growth over time curves and we propose a novel semi-mechanistic model capable of describing the non-monotonic growth including the interaction between cancer cells, angiogenesis, and resources such as nutrients or oxygen, in the tumor microenvironment. Our model, with respect to previous literature models, improves diagnostic plots such as weighted residuals versus time plots and residuals lag plots, and individual predictions. Additionally, the framework allows the incorporation of anticancer treatments considering their mechanisms of action. Therefore, the model constitutes a valuable tool in the development of therapeutic strategies, supporting the rational design and selection of different treatment scenarios in preclinical drug development.

## Introduction

Tumor growth dynamic (TGD) modeling represents a key element of model-based drug discovery and development in oncology [1]. Specifically, among other applications, it has been used to select promising drug candidates, assist the selection of the first-in-human dose, generate predictive quantitative translational frameworks, leverage clinical data, and identify relevant biomarkers [2–5].

**Fig 1:**
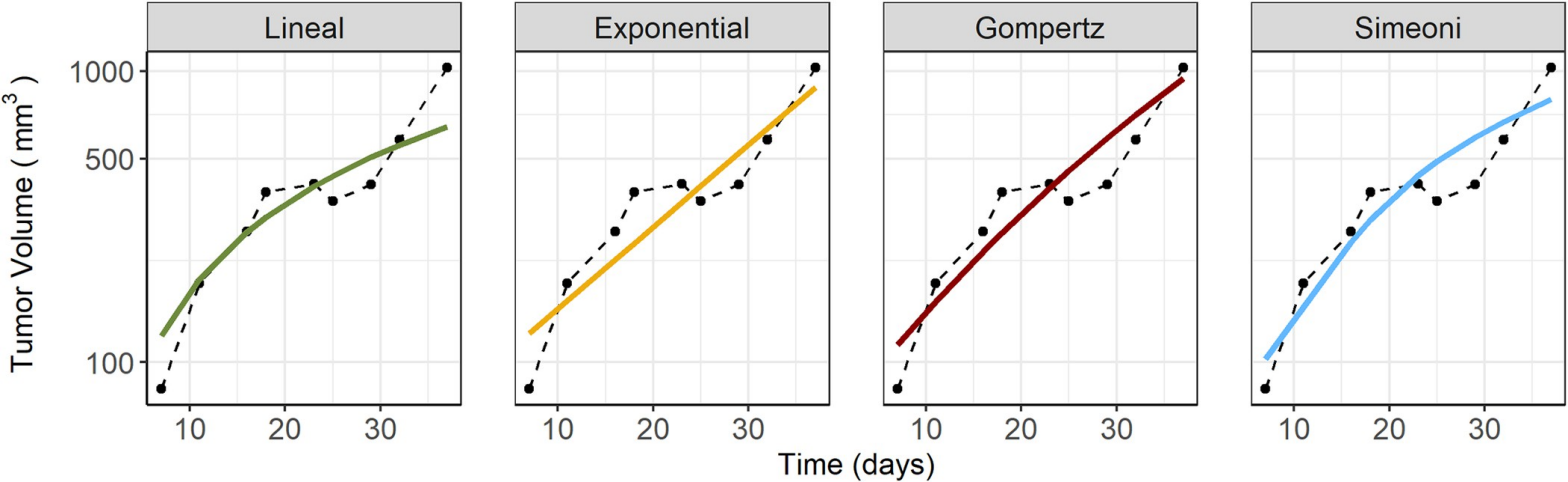
Tumor volume over time of a single mouse from a control group which was inoculated with lung H1975 tumor cells. The raw data (observations represented with points and joined with dashed lines) shows an apparent oscillatory profile in contrast with the predictions obtained from the commonly used tumor growth dynamics models (solid colored lines).

Over the years, different models have been developed to describe tumor progression and tumor shrinkage effects of different anticancer therapeutic strategies [6–9]. From a high-level perspective, these tumor growth inhibition (TGI) models incorporate two main components: (i) disease (unperturbed tumor) progression and (ii) treatment effects. The latter comprises all aspects driving the link between drug exposure and drug effects. On the other hand, disease progression models describe the natural growth of cancer cells in the absence of treatment [10]. Currently, different model structures have been proposed to characterize unperturbed tumor dynamics relying on reasonable assumptions and providing adequate descriptive and predictive power [11–13] (see methods section for a detailed description of the most used models in data-driven analysis). The main characteristic of these model structures is their monotonic nature (i.e. predictions are entirely non-increasing, or entirely non-decreasing). This is due to the fact that most currently accepted models are variants of the exponential model with scarce mechanistic support. However, data from the literature indicate that, even in the absence of treatment, tumor growth rate may increase or decrease at different stages generating oscillatory patterns [13–15], which cannot be satisfactorily described by the aforementioned models (Fig 1).

Tumor growth is a complex and dynamic process involving cell-cell and cell-extracellular matrix interactions that allow cancer cells proliferation, drug resistance and metastasis. During the development of solid tumors, a large amount of nutrients is consumed due to the rapid proliferation of tumor cells [16]. Moreover, high oxygen consumption, lack of nutrients and accumulation of metabolic substances in cells can create an oxygen-deficient microenvironment that is not suitable for tumor cell growth [17]. Tumor hypoxia-induced responses include, among others, enhanced angiogenesis and vasculogenesis [18]. Moreover, hypoxia also contributes to a reduced anti-tumor immune response through different pathways [19]. These processes can result in patterns of growth showing deceleration followed by acceleration (oscillatory tumor growth patterns).

Mathematical model structures can be constructed to reveal key mechanisms generating subtle states of imbalance that could explain the oscillatory patterns in tumor growth. However, to the best of our knowledge, limited mathematical models are available describing these types of dynamics and (i) including the interplay of the multiple elements forming the tumor microenvironment (TME) in a mechanistic fashion, (ii) using a few sets of ordinary differential equations, and (iii) estimating precise model parameters in absence of anticancer treatment.

From a theoretical perspective [20–22], very recent reports suggest that the prey-predator concept, largely used in ecology, could resemble the interaction between cancer cells and immune cells [20–22]. And, although the dynamics of immune cells and target cells present some discrepancies with the classical ecological framework, it inspires a new perspective on the possible mechanisms involved in tumor progression. Particularly, the work carried out by Kareva et al. [20] proposes a prey-predator-based model to describe the interplay between a heterogeneous population of tumor cells (prey) and cytotoxic immune cells (predator), and compares different types of interactions (i.e. mutualism, competition, prey-predator), with the multiple mechanisms undertaken in the TME. However, results are based on multiple simulations using literature parameters and do not include experimental observations supporting the theoretical framework.

The development of model structures capable of characterizing non-monotonic behaviors of tumor growth is highly relevant, particularly at the preclinical stage, since due to its translational capacity, a more accurate estimation of the parameters that govern cancer cells proliferation could necessarily affect the design of dosage schemes or treatment combinations that guarantee optimal efficacy results.

The objective of the current investigation is to develop a data-driven model characterized by a structure describing oscillatory tumor growth profiles and untangling the underlying mechanisms in untreated mice. To fulfil that objective, xenograft-derived unperturbed tumor growth longitudinal data obtained from a large set of tumor cell lines and several cancer types were analyzed using the non-linear mixed effect approach. As a corollary, the capability of the model to incorporate the effect of different treatments is also explored.

## Materials and methods

### Ethics statement

All the experiments were approved by the Eli Lilly and Company institutional Animal Care and Use committee.

### Experimental data

Data from control (unperturbed) groups of different xenografts preclinical studies were pooled together, resulting in longitudinal tumor volume (TV) data from 85 mice (individual volume measurements = 1,064), including 12 different cell lines from 6 tumor types (breast (MB-321), leukemia (MV411), lung (A549, Calu-6, H1650, H1975, H2122, H441), lymphoma (JEKO-1), melanoma (A2058, A375) and pancreas (MIA PaCa-2)). Additional details regarding the number of animals and observation in each cell line can be found in Table A in S1 Text. Moreover, a comprehensive description of the experimental procedures can be found elsewhere [23]. Briefly, after subcutaneous inoculation of human-derived tumor cells into the flank of athymic nude mice, tumor size was measured using a caliper. When the tumor exceeded the upper tumor size limit defined in the ethical protocol, mice were sacrificed. Tumor volume (TV) was then computed assuming an ovoid form: (length × width^2^)/2 [24].

### Empirical exploratory analysis of tumor volume profiles

**Fig 2:**
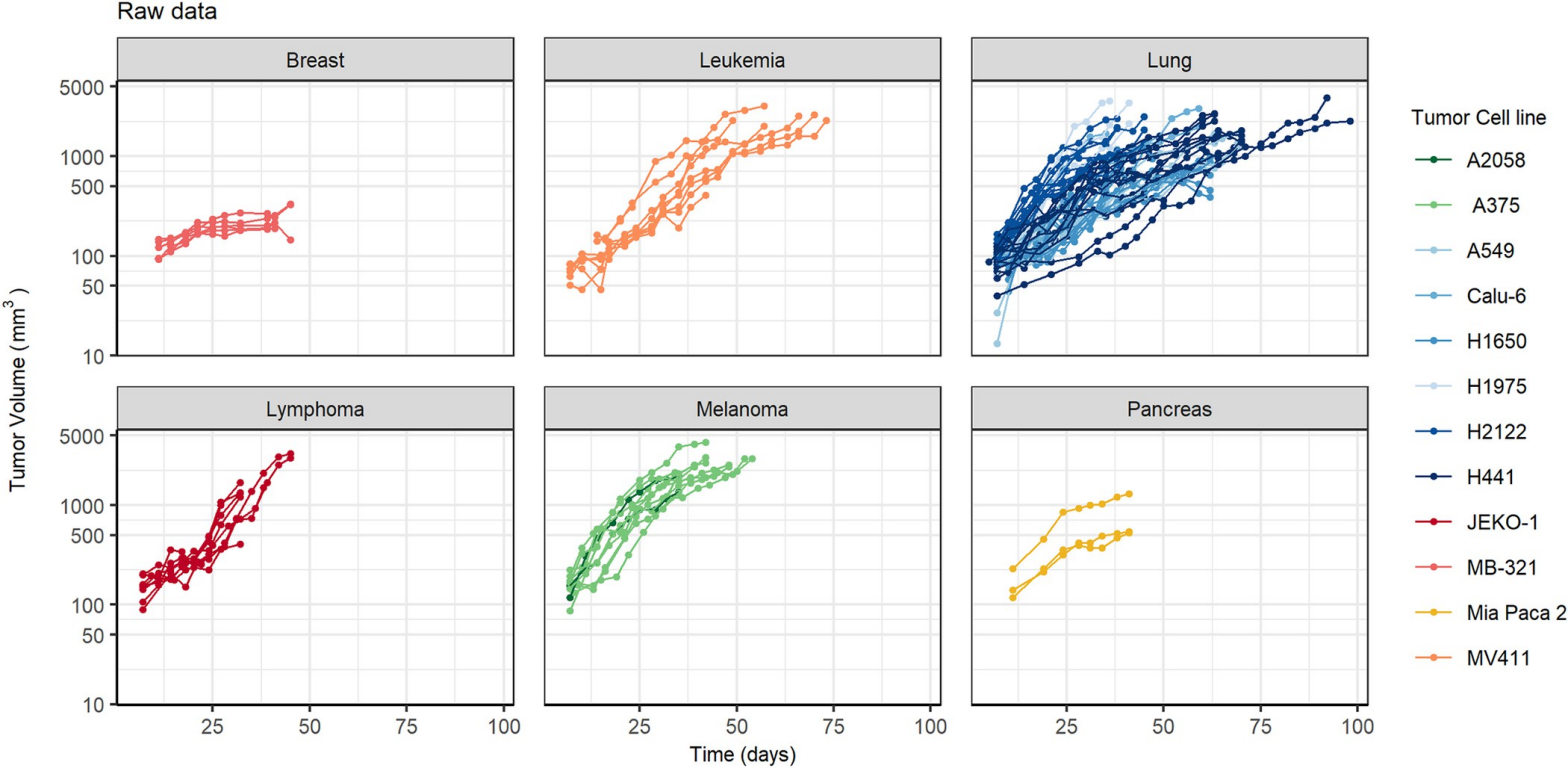
Tumor volume raw data from multiple experiments including different tumor types (panels) and cell lines (different colors in each panel). Points and colored lines represent the observed tumor volume observations for each individual.

Tumor volume observations (Fig 2) were first analyzed empirically to characterize the oscillatory behavior and investigate if the patterns were systematically observed across the different tumor types and cell lines.

**Fig 3:**
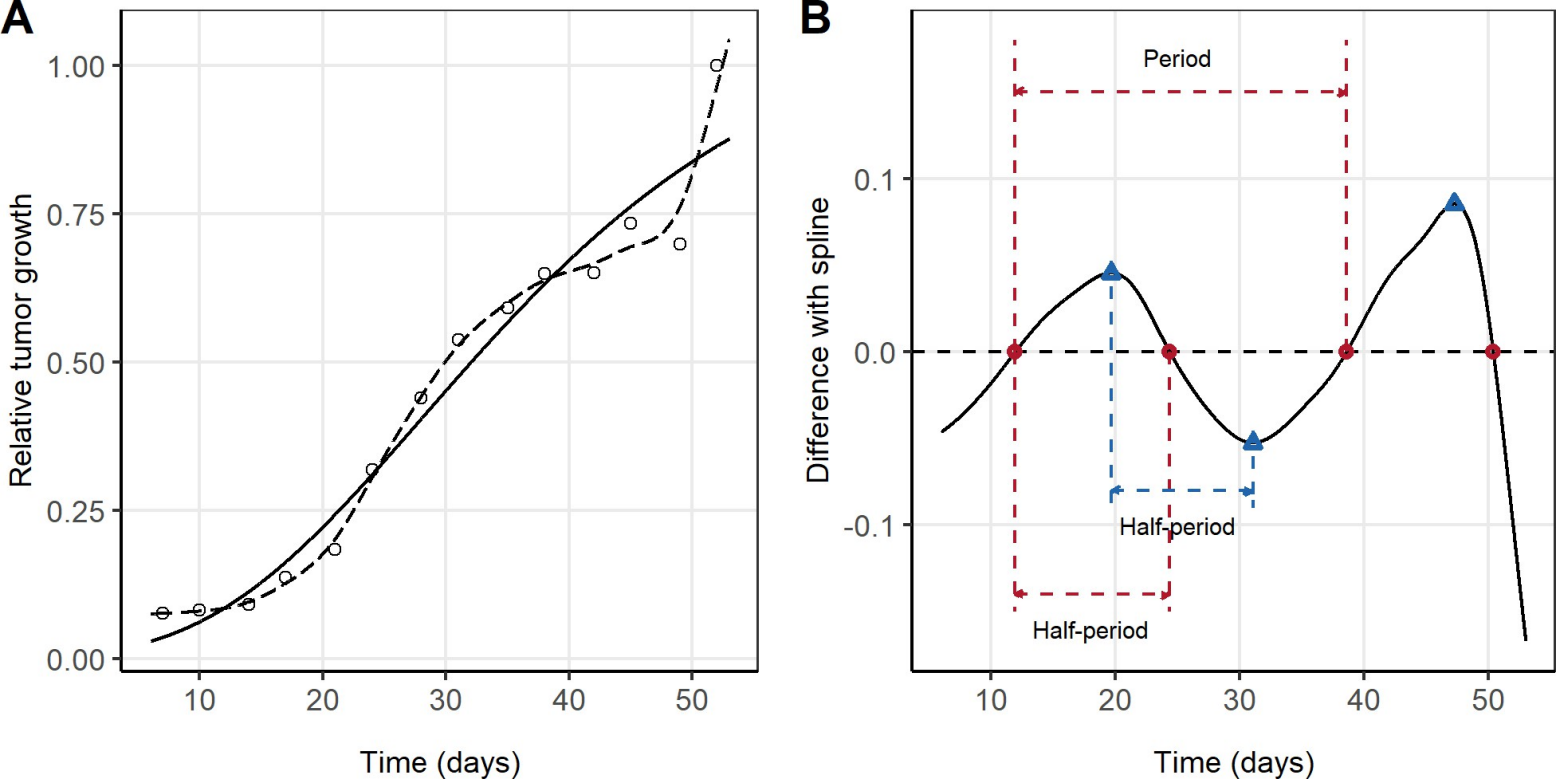
(A) Single case example of tumor volume observation (dots) fitted with the spline function (dashed line), and with a classical tumor growth dynamic model, in this case Gompertz equation (solid line). (B) Using the spline line of the case example as reference (dotted black line), the difference with the best classical model for each individual (Gompertz equation for this case example) is represented with a solid black line. Additionally, crossing with the zero-line (red points) and the local extrema (blue triangles) are marked in the graph.

For this purpose, raw data were first transformed in two different ways aiming for a fair comparison between different individuals: (a) logarithmic transformation of TV (log transformation) and (b) rescaling of the TV with the maximum observed growth for each animal (unit normalization transformation). Then, two different approaches were used to fit the raw data profiles: (i) classical TGD models (i.e. linear, exponential, Gompertz or Simeoni) using non-linear regression and (ii) the cubic natural spline function using smoothing spline function ([curve, gof_spline,out_spline] = fit (t, f, ’smoothingspline’)) implemented in MATLAB version 9.10.0 (R2021a) (The MathWorks Inc.) [25]. The best-performing classical TGD model for each individual according to the adjusted R^2^ value was selected. Fig 3 presents a particular individual as to case-example the analysis in which the computed adjusted R^2^ for the exponential, logistic, Gompertz and Simeoni were 0.90, 0.95, 0.96, and 0.95, respectively, whereas for the cubic spline was 0.98. Fig 3A presents tumor volume observations (dots) of the case example, together with the spline function fit (dashed lines) and the best-performing classical TGD model (Gompertz) (solid line).

Subsequently, the difference between the classical TGD model fit and the spline function (used as reference) was computed and used to study the individual oscillations. For the case example used in Fig 3, panel B described the difference between both models (blue solid line), together with the zero-crossings (crossing with the zero-line–red dots), and extrema (maximums-blue dots and minimums-green dots). Additionally, the figure also reflects the oscillatory periods and half-periods. Specifically, the oscillatory half-period (HP) (time needed to complete half-oscillation) was used as the selection metric

To further address the question of whether the perceived oscillations are artefacts due to inherent noisy data or true oscillating signals a simulation study was performed. For each mouse data (unit transformation), we first applied the best-performing classical TGD (either linear, exponential, Gompertz or Simeoni) fit and then perturbed such fit with a Gaussian white noise with the same variance as the raw data. Subsequently, we performed 5,000 simulations of each individual and computed the corresponding half periods (HP) in the same way as we did during the exploratory data analysis stage. The distribution of the exploratory and simulated HP was visually compared using a histogram. Additionally, the percentiles of HP obtained in the exploratory stage with respect to the simulated data were computed and the Kolmogorov-Smirnov (KS) test was used to evaluate whether they were uniformly distributed. A more detailed description can be found in S1 Text.

### Non-linear mixed effects modeling of tumor growth dynamics

Population analysis was performed using the nonlinear mixed-effects modeling (NLME) [26] approach. Specifically, population parameter estimation was performed using the SAEM (stochastic approximation expectation minimization) algorithm coupled with the Markov Chain Monte Carlo procedure implemented in Monolix 2021R1, Lixoft SAS, a Simulation Plus company [27].

During model building, data were fitted to the different tumor growth dynamic models: classical TGD models and tumor growth models integrating biological. Additionally, the full set of TV vs time profiles was analyzed simultaneously and using the log transformation. Inter-individual variability (IIV) was modeled exponentially, and residual error was described using an additive error model on the logarithmic scale.

### Unperturbed tumor growth models

a. Classical tumor growth models

The linear, exponential, Gompertz and Simeoni models were fitted to the longitudinal TV data in the absence of anticancer treatment (Table A in S2 Text). The list of explored models does not intend to be exhaustive but does represent the most used ones. All of these models resemble just the growing mechanism and share certain common features as the initial condition (predicted tumor volume after cell inoculation (TV_0_)), and the capability to estimate the full set of model parameters in a data-driven modeling exercise. However, what is relevant to the current investigation, is that the model structures predict a monotonic (smoothly continuous) increase in tumor growth.

b. *Tumor growth models integrating biological mechanisms*

The presence of oscillatory behavior in the tumor volume versus time profiles motivated the use of models representing different aspects of the complex biological system such as angiogenesis, the role of the immune system, and tumor heterogeneity. The data were also fitted to tumor growth models including biological mechanisms (Table B in S2 Text).

However, since none of the models explored so far provided a fair description of the data a novel model structure was sought. Our proposed model assumes that cancer cells will both consume resources (RES) and promote angiogenesis (ANG) in order to increase the amount of RES delivered to the TME. Additionally, the model accounts for cancer cells and blood vessels’ natural degradation.

**Fig 4:**
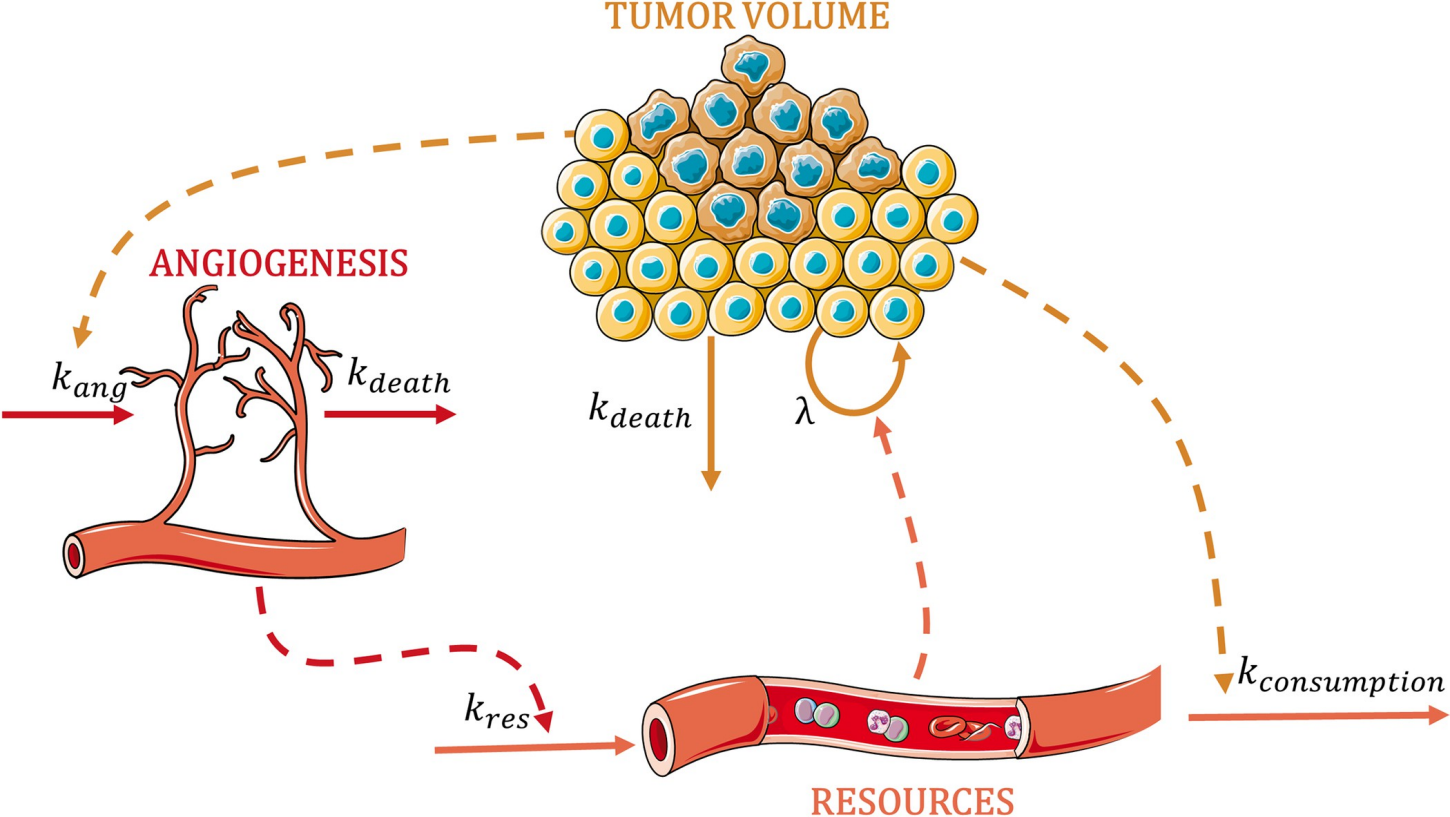
Schematic representation of the final model. Solid arrows indicate the proliferation/activation or the death processes, and the dashed arrows describe the different interactions between the three species. Parameters are defined in the text. Graphical elements are adapted from Servier medical art repository (https://smart.servier.com).

Fig 4 describes schematically the final selected model which is mathematically represented by the following set of differential equations:

$$\frac{dTV}{dt}=\lambda \times TV\times RES-k_{death}\times TV$$ (1)

$$\frac{dANG}{dt}=k_{ang}\times TV-k_{death}\times ANG$$ (2)

$$\frac{dRES}{dt}=k_{res}\times ANG-k_{consumption}\times TV$$ (3)

where the growth rate is governed by the second-order rate constant λ; k_ang_ and k_res_ are the first-order rate constants governing the proliferation of angiogenesis and resources, respectively; k_death_ is the first-order rate constant representing natural degradation, and k_consumption_ first-order rate constant reflects the consumption of resources by cancer cells. Note that tumor volume was measured in mm^3^ while ANG and RES are expressed as arbitrary units (au). In addition, tumor volume at the time of cell inoculation (TV_0_) is a parameter of the model to be estimated, however, ANG and RES are initialized at a value of 1.

Once the final model, described by Eqs 1–3, was selected based on the model selection criteria (see below), a mathematical and numerical analysis was undertaken to understand the integrated dynamics of the different entities of the model (tumor, resources, angiogenesis). For this purpose, the equilibrium points and the dynamic behavior of the system were investigated (in section C of S2 Text).

### Model selection and evaluation

Selection between competing models was performed using the (i) Akaike information criterion (AIC) which applies a penalty proportional to the number of model parameters, (ii) precision of parameter estimates expressed as the relative standard error (RSE %) calculated as the ratio (multiplied by 100) between the standard error and the parameter estimate, and (iii) visual inspection of the goodness-of-fit (GOF) plots including residuals lag plots [28].

In addition, simulation-based diagnostics, and visual predictive checks (VPCs) were generated to evaluate further the ability of the model to resemble both the typical tendency and dispersion of the data. In brief, 1,000 datasets of the same characteristics as the original one was simulated using the selected model and its parameter estimates. Resembling the experimental design, tumor size values above the prespecified upper limit [26] were removed from the simulation. Then, for each simulated dataset, the 2.5^th^, 50^th^, and 97.5^th^ of the TV were calculated per time interval, and the 95% prediction intervals of the aforementioned percentiles were graphically superimposed onto the 2.5^th^, 50^th^, and 97.5^th^ percentiles of the raw data.

Finally, the relevance of the different model parameters on the system was evaluated in a local sensitivity analysis. One parameter at a time was increased or decreased by 50% and subsequently, tumor volume, the amount of resources and the amount of angiogenesis at day 20 were computed for each scenario. The selected time for the sensitivity analysis reflects the time after cell inoculation at which tumor volume was approximately half of the maximum allowed by the ethical committees.

### Model exploration

Deterministic simulations in which different values of k_consumption_, k_ANG_, and k_RES_ were used to further explore the effect of anticancer treatment on tumor shrinkage. First, the parameters were increased or decreased by 25% and 75% and the tumor volume, angiogenesis and resources over time curves (from day 0 to day 100) were obtained for each scenario. In addition, to simultaneously evaluate the impact of parameters on the model outcome (i.e. area under the tumor volume versus time curve (AUTC) until day 100), 200 different combinations using Sobol’s method [29] as approximated by Saltelli and colleagues [30] and implemented in SAFE toolbox R package [31] were obtained. The most relevant parameters according to the sensitivity analysis (k_consumption_, k_ANG_, and k_RES_) were sampled from the (plausible) parameter space using the Latin Hypercubic method and assuming a uniform distribution in the log domain.

## Results

### Empirical exploratory analysis of tumor volume profiles

**Fig 5:**
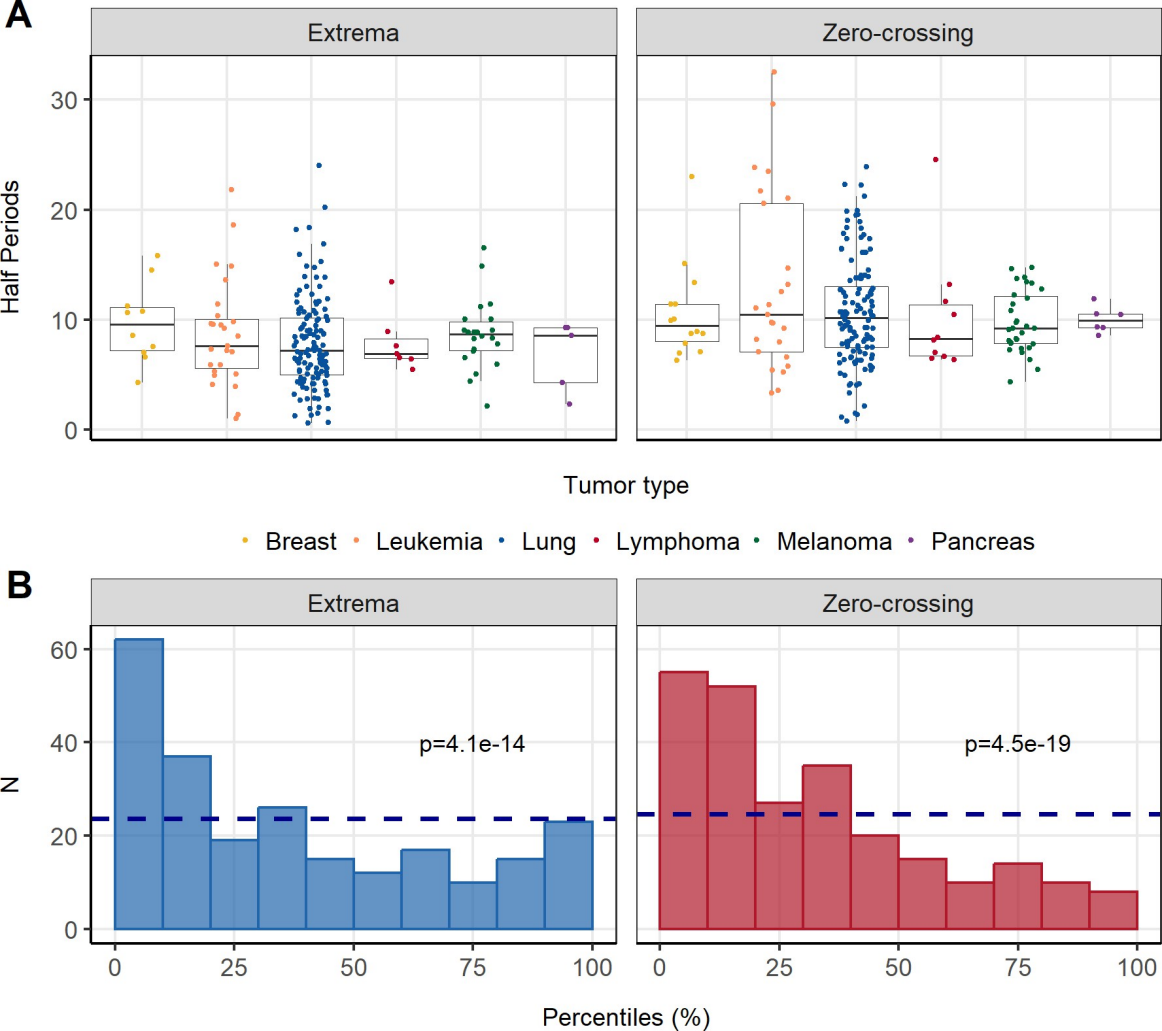
(A) Boxplots of the half-periods for the different tumor types. (B) Estimated percentiles and p-value of Kolmogorov-Smirnov (KS) test for zero-crossing (blue) or extrema (red). The dashed blue lines correspond to the expected height of all the bins for the uniform distribution.

The oscillations of our experimental data were characterized using the exploratory analysis showed for a single mouse in Fig 3. Across the different tumor types, similar oscillatory HP distributions were observed, with a mean between 8 and 11 days (Fig 5A).

Moreover, the simulation study (see S1 Text for a complete description of the different steps using a single case example) confirms that the observed HP are extreme with respect to the distribution of the simulated HP. Therefore, suggesting that the oscillations are a landmark of the underlying mechanisms driving tumor growth and not part of the residual error. Specifically, the results show, both graphically (Fig 5B) and statistically (extremely low p-value of the KS test) that the estimated proportions of simulated HP above the experimental HP are not homogenously distributed and thus not random.

### Non-linear mixed effects modeling of tumor growth dynamics

Unperturbed Tumor growth models

a. *Classical tumor growth models*

Out of the models listed in Table 1, the Simeoni model [32] provided the best description of the data and was associated with the lowest AIC value. The difference in AIC with respect to the Simeoni model was 1412, 370, and 209 for the linear, exponential and Gompertz models, respectively. The final parameter estimates for each model and the AIC values can be found in Table A in S2 Text. The visual predictive check of S3 Fig demonstrates an adequate performance of the Simeoni model. However, when studying the weighted residuals over time (S3 Fig) an oscillatory pattern up to day 50 can be observed. In addition, S3 Fig further evaluates model performance through the autocorrelation plot. The lag plot indicates a significant autocorrelation between residuals (p<0.001), suggesting that the tumor dynamics are not fully described by the model structure.

b. *Tumor growth models integrating biological mechanisms*

The different models explored in section B in S2 Text describe different entities of the TME and mechanisms involved in cancer progression. The aforementioned models including the immune system [33,34], cancer cells heterogeneity [6,35], or angiogenesis (carrying capacity dynamics) [14,36] were unable to reproduce an oscillatory pattern. Therefore, the complex behavior of the raw data in unperturbed conditions was not captured (see the typical profile of each model in Table B in S2 Text).

**Fig 6:**
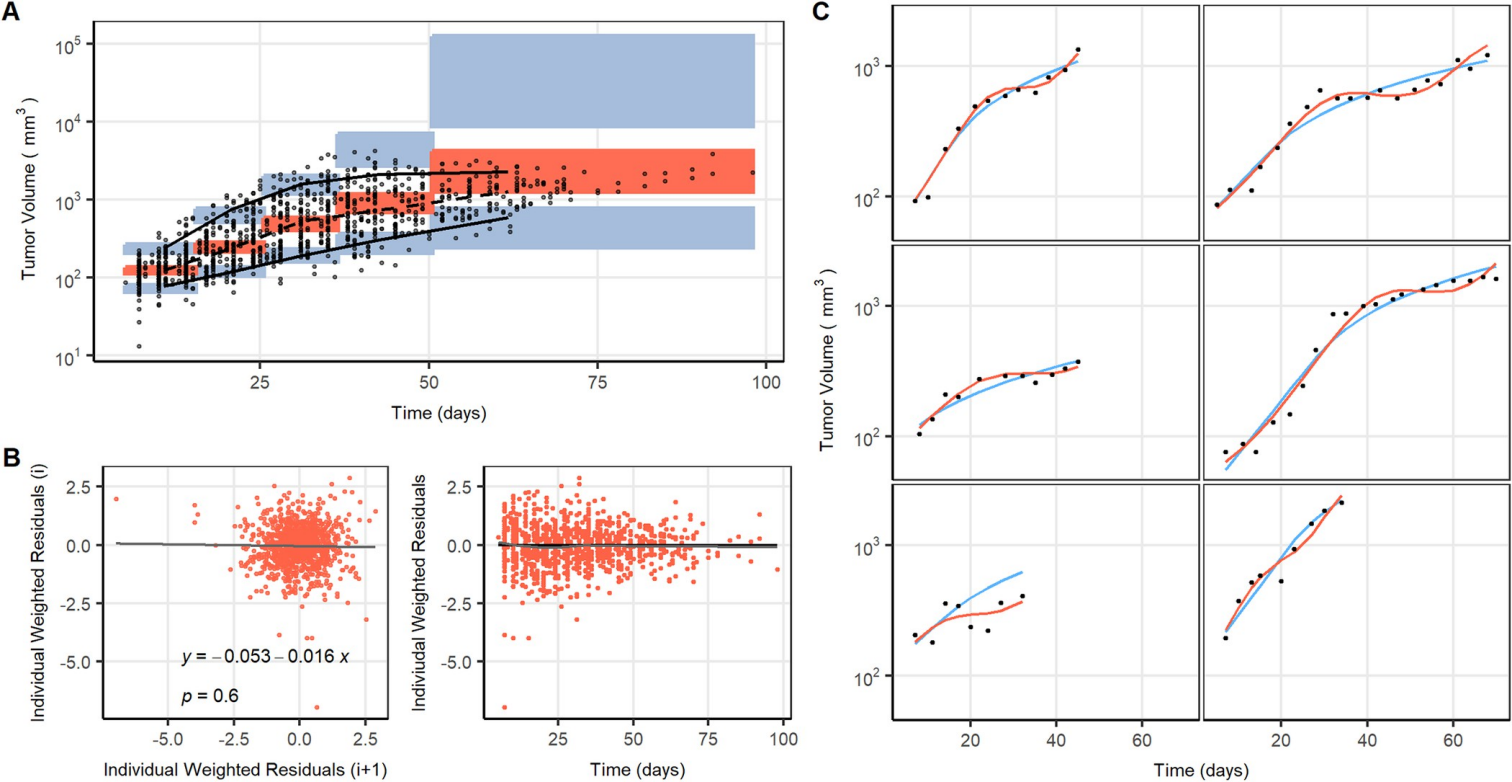
Models results and a graphical evaluation of the final tumor growth model: (A) visual predictive check: the black dots show the tumor volume measure, black lines represent the 5^th^, 50^th^ and 95^th^ percentiles of the raw data, colored areas denote the 95^th^ confidence interval of model-predicted median (orange areas), 5^th^ and 95^th^ percentiles (blue areas). (B) Weighted residuals versus time (right panel) and lag plot (where i represents each residual value in chronological order of observations) (left panel). (C) Tumor volume vs time observations (solid circles) and individual model predictions corresponding to the individual model parameters obtained from the selected (orange) and Simeoni (blue) models, for six different mice chosen at random.

The novel tumor growth model described by Eqs 1–3 and represented in Fig 4, provided an adequate precision for parameter estimates (RSE <30%) and a proper description of the TV profiles (Fig 6A). With regard to random effects, our data supported the inclusion of inter-individual variability in all the parameters except k_res_ whose IIV was associated with model instabilities. Moreover, although no treatment is administered, the model supported the inclusion of tumor cell death (naturally occurring) and blood vessel degradation, both processes governed by a k_death_. The final model parameters can be found in Table 1.

**Table 1:** Final model parameters of the tumor growth model.

TG Model	Prey-predator	
AIC		
	122.97	
Parameters		
	Estimate (RSE%)	IIV (RSE%)
TV_0_ (mm^3^)	69.06 [4]	0.29 [10]
λ (au^-1^×day^-1^)	0.046 [6]	0.44 [8]
k_ang_ (au× mm^-3^× day^-1^)	0.00036 [5]	0.31 [11]
k_res_ (day^-1^)	0.11 [4]	
k_consumption_ (au× mm^-3^ ×day^-1^)	0.00069 [5]	0.35 [10]
k_death_ (day^-1^)	0.0081 [7]	0.27 [23]
Residual error (log(mm^3^))	0.17 [3]	

AIC: Akaike information criteria; IIV: inter-animal variability expressed as coefficient of variation $\left(\sqrt{e^{\omega^{2}}-1}\right)$; RSE: relative standard error in percentage (%)

The results of the novel structure were significantly an improvement over the previously developed models. This was testified by the GOF plots (Fig 6B); both the weighted residuals versus time and the lag of autocorrelation observed in the lag plots (p>0.05), not forgetting the good quality of the individual predictions (Fig 6C). Additionally, a lower AIC and a decreased error were obtained compared to the Simeoni model (ΔAIC = -56.24 and Δresidual error = -0.02).

**Fig 7:**
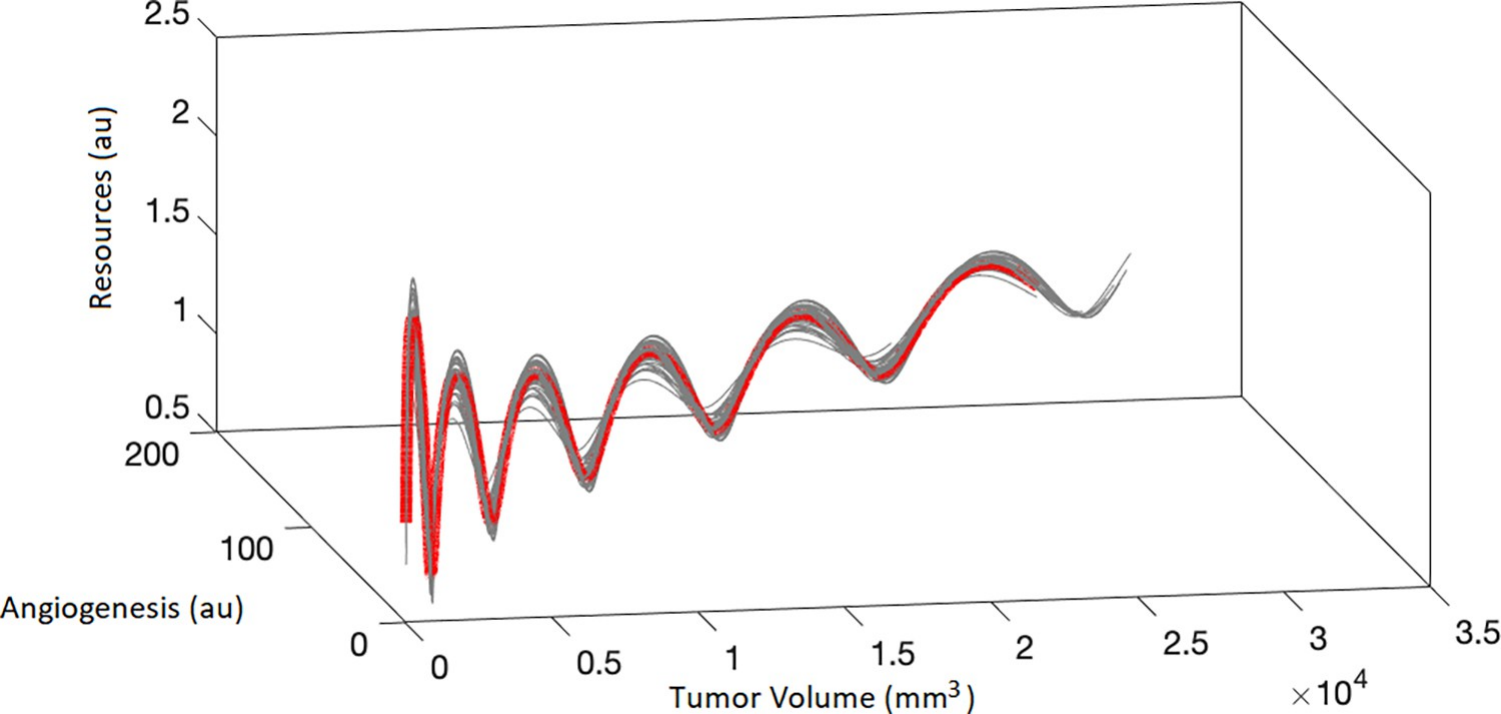
Numerical solution of the mean population parameters (red thick curve), together with 50 solutions (gray lines) obtained by perturbing the initial conditions by a Gaussian white noise vector (the variance for resources, angiogenesis and tumor volume being 0.1, 0.1 and 1, respectively). The plot reflects the dynamics of the resources (au) (RES), angiogenesis (au) (ANG), and tumor volume (mm^3^) (TV).

The mathematical and numerical analysis (see section C in S2 Text) revealed that the system, given the final parameter values (Table 1), has only one equilibrium point (the trivial one) with no eigenvalues associated. Nevertheless, the numerical integration shows that the system exhibits an oscillatory behavior. Fig 7 depicts the numerical solutions for the final model parameter estimates and 50 other simulated solutions resulting from slightly perturbed initial conditions. Additionally, the behavior observed in Fig 7 highlights two important aspects regarding the dynamics: (i) that the solution is oscillatory with non-constant frequency and (ii) that the initial condition perturbations lead to very similar solutions, showcasing the robustness of the population solution.

**Fig 8:**
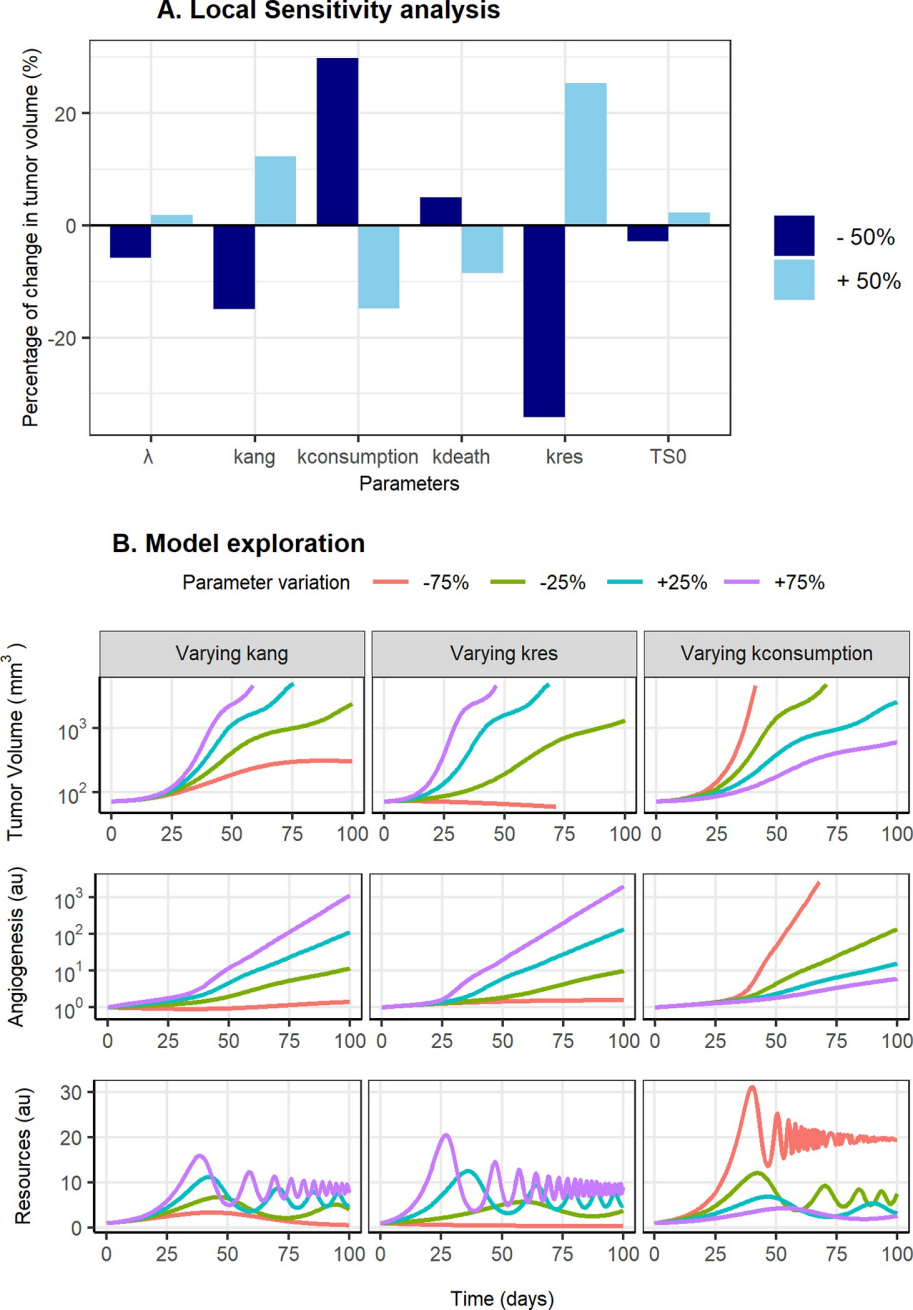
Sensitivity analyses. (A). Sensitivity analysis of the model when one parameter at a time is modified representing the percentage of change in tumor volume at 20 days after tumor inoculation. B. Deterministic model simulations of the tumor size, angiogenesis and resources over time for different values of k_ang_, k_res_ and k_consumption_.

Following the exploration of the model, the sensitivity analysis (Fig 8A) reflects that TV is more sensitive to the parameters governing the proliferation (k_res_) and consumption (k_consumption_) of RES. And, in a smaller extent, to the proliferation rate constant of angiogenesis (k_ang_). TV at baseline (TV_0_) and λ are the parameters that least influence the final TV observed. Additionally, the deterministic simulation (Fig 8B) performed after varying the aforementioned parameters show that higher values of k_res_ and k_ang_ or lower values of k_consumption_ resulted in a higher oscillatory frequency of RES, and thus in an increased TV. Moreover, although tumor shrinkage is only achieved by decreasing k_res_ 75% (red-Fig 8B), the results suggest that a slower tumor growth will be observed with different scenarios. Including among others, anti-angiogenesis treatments (decreasing k_ang_), resources proliferation inhibition (decreasing k_res_), cancer cells killing or blood vessels degradation (higher values of k_death_), and elimination of TME resources (increasing k_consumption_). Due to this, in order to observe a reduction in TV, different anti-cancer treatments might need to be combined. In fact, currently, successful anticancer therapies are based on different combination approaches [37].

**Fig 9:**
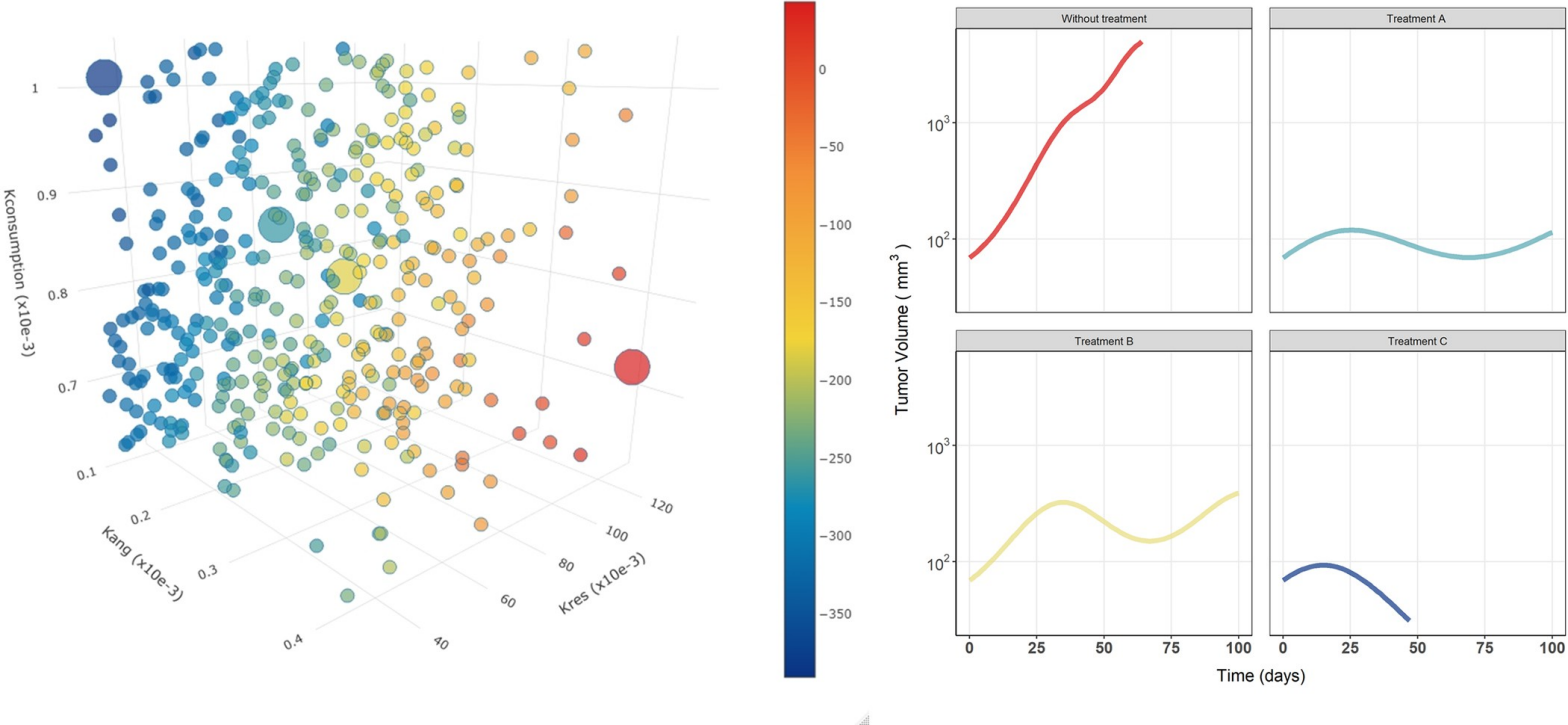
Impact of virtual combination therapies. Points represent the change in the area under the tumor size vs time curve (AUTC) for each combination of the k_res_, k_ang_, and k_consumption_ parameters with respect to the one corresponding to the typical parameter estimates (Table 1). The circle in red corresponds to the reference AUTC value. AUTC was calculated from time 0 to 100 days. Additionally, a tumor volume profile over time for four different parameter combinations is shown on the right side of the figure.

Fig 9 provides an overview of a wide range of parameter combinations which can potentially result from different treatment combinations. The difference in the AUTC of each simulated scenario with respect to the typical individual (without treatment–individual colored in red in Fig 9), was used as the selection metric. The combination of different antiangiogenic drugs affecting both k_ang_ and k_res_, (Fig 9 -treatment A and B) results in a decreased tumor growth rate, resembling the tumor volume profiles of stable disease or partial responder patients. On the contrary, the cytotoxic effects of chemotherapy result in an initial decay not observed in the previous scenario. Moreover, when either chemotherapy or immunotherapy (promoting cancer cell death or blood vessel degradation) (k_death_) is combined with an anticancer treatment able to inhibit tumor resources, the highest tumor volume reduction (Treatment C of Fig 9 presents the lowest AUCT). In summary, the model reflects the high variability in the response observed in preclinical and clinical scenarios and suggests that combination strategies are needed to succeed in cancer treatment.

## Discussion

The main contribution of this work is the development of a simple but mechanistic based tumor growth model that retrieves precise model parameter estimates using only tumor longitudinal observations gathered from standard experimental settings in unperturbed mice. Furthermore, although the Gompertz expression has demonstrated a high predictive power [11,13] and adequately fits both preclinical [12,38] and clinical data [39], the proposed model showed increased adequacy in all the numerical and graphical diagnostics performed. In this respect, the lag plots, scarcely used as a goodness of fit plot to compare among competitor models, emerge as a powerful diagnostics tool to support the selected model.

The development of this novel tumor growth model was motivated by the oscillatory patterns observed in unperturbed (i.e., in the absence of anti-cancer treatment) tumor growth profiles obtained in a variety of xenograft studies. The new model proposal describing non-monotonic growth assumes: (i) angiogenesis induced by cancer cells, (ii) the increase of tumor growing resources (i.e. nutrients and oxygen) as a result of angiogenesis, (iii) tumor growth dependent on the available resources, and (iv) consumption of resources by cancer cells.

Remarkably, the possibility that those patterns were the consequence of random variations was discarded based on the results of a statistical analysis performed comparing the distribution of random HP oscillation and the observed HP (KS test with a p-value lower than 10^−9^). In addition, the statistical analysis also revealed that the characteristics of the oscillations were evenly distributed across the different cell lines. No pattern suggesting that a particular tumor type was more prone to show non-constant growth rates was observed. It is also important to note that, although a formal analysis was not performed, intuitively, we can conclude that in those cases where there is a sparse sampling, the detection of the oscillatory behavior might be jeopardized. Therefore, highlighting the relevance of the study design and the sampling schedule to identify the optimal times for tumor volume measurements that will inform as maximum as possible the different model parameters within a given model structure [23,40].

Even though different authors have pointed out that even in the absence of treatment, tumor growth rate can vary over time as a consequence of the TME [20,41,42], limited mathematical models have been developed characterizing this complex dynamic. There are a few models describing oscillatory tumor growth profiles in untreated groups through complex mathematical frameworks or from a more theoretical approach [41–44]. However, interestingly, the vast majority of models that are being currently used to describe the aforementioned profiles using the non-linear mixed effects approach treat the deviations around the monotonic growth as noise (residual error). The oscillatory patterns are then described as a result of treatment administration, and once the treatment is rescinded, tumor growth returns to follow a monotonic increase.

Thus, even though our work is not the first attempt of studying this complex pattern, to the best of our knowledge, it is the first data-driven modelling exercise that describes non-monotonic tumor growth profiles in the absence of treatment through a simple system of ordinary differential equations, easily implemented in any modelling platform. Nevertheless, note that one of the limitations of this structure is that only tumor volume measures were available and therefore, no experimental data were available to confirm the mechanisms behind the oscillatory patterns. On the one hand, systemic biomarkers reflecting the multiple mechanisms taking place in the tumor microenvironment are hardly available as longitudinal observations. And on the other hand, complex designed experiments including the administration in monotherapy and in combination with multiple anti-cancer drug acting on different mechanisms will be needed in order for the tumor volume data to reflect tumor growth mechanisms.

In our model, the initial tumor volume (TV_0_) and the tumor growth rate constant (λ), estimated to 69.06 mm^3^ and 0.046 day^-1^, respectively, were within the range obtained with reference models. This highlights the fact that although the classical TG models are not able to capture the oscillatory behavior, they are generally capable of describing the mean trend of the data. In addition, the previous publication [23] in which the same experimental data was analyzed using the Simeoni model suggested that certain model parameters were different across cell lines. However, in the current work, although we investigated the different HP for each tumor type, we did not consider these differences during model development. The present analysis should be extended in order to further study the non-monotonic pattern in each tumor type or cell line.

With the proposed model, which includes key biological mechanisms involved in tumor progression, emerges the possibility of locating drug effects more mechanistically on different parameter targets. Figs 8 and 9 of the main text suggest that both treatments which decrease k_res_ and k_ang_ or increase k_consumption_ and k_death_, present slower tumor growth profiles or even show tumor shrinkage. Therefore, the importance of this approach lies in its potential translational impact on drug combinations from different angles. Even in more mechanistic models, the drug effect is only included in tumor death [7].

Furthermore, the aspect of optimizing the dosing schedules in combination therapies remains elusive. Increasing the mechanistic understanding of the system gives the opportunity to explore whether different dosing schemes can increase the benefit and lower toxicity. One example of hematological toxicity in chemotherapy is the model built for diflomotecan [45] which includes the coexistence of different states of cancer cells (proliferative and quiescent).

In summary, the work presents a new semi-mechanistic model able to describe the non-monotonic growth and the interaction between the tumor, angiogenesis and resources in the TME. This framework constitutes a valuable tool to explore different mechanisms of action in a more mechanistic fashion and thus supporting the rational design and selection of drug candidates in different scenarios.

## Supporting information

S1 Text Raw tumor volume data and empirical exploratory analysis of tumor volume profiles. (DOCX)

S2 Text Tumor growth dynamic models. (DOCX)

S1 Fig Graphical representation of the experimental half-periods (HF). (TIF)

S2 Fig Histogram of the simulated half-periods (HP) (5000 simulations) calculated using the crossing with the zero-line (left) and the local extrema (right). The total number of calculated HP from the simulations are N = 24, 126, and N = 24,770, for the zero-crossing and extrema method, respectively. The symbols in each panel indicate the evaluated HPs corresponding to one subject (ID = 1066) of the original data. For this specific subject, we indicate the proportion of simulated data that les above the given original HP.
(TIF)

S3 Fig Models results and a graphical evaluation of the Simeoni tumor growth model: (A) visual predictive check: the black dots show the tumor volume measure, black lines represent the 5th, 50th and 95th percentiles of the raw data, colored areas denote the 95th confidence interval of model-predicted median (orange areas), 5th and 95th percentiles (blue areas). (B) Weighted residuals versus time. (C) Lag plot (where i represents each residual value in chronological order of observations).
(TIF)

S4 Fig Numerical integration of the differential equations describing the tumor growth. The parameters of the equations are taken from Table 1. Panel (a) shows the 3D phase space, the red curve corresponds to the main solution and the superimposed 50 thin gray lines correspond to the slightly perturbed initial conditions. Panels (b-d) correspond to the 2D projections of the solutions shown in panel (a).
(TIF)

## References

[1] Al-Huniti N, Feng Y, Yu J, Lu Z, Nagase M, Zhou D, et al. Tumor Growth Dynamic Modeling in Oncology Drug Development and Regulatory Approval: Past, Present, and Future Opportunities. Cit CPT Pharmacometrics Syst Pharmacol. 2020;9:419–27. doi: 10.1002/psp4.12542 32589767 PMC7438808

[2] Lindauer A, Valiathan C, Mehta K, Sriram V, De Greef R, Elassaiss-Schaap J, et al. Translational Pharmacokinetic/Pharmacodynamic Modeling of Tumor Growth Inhibition Supports Dose-Range Selection of the Anti-PD-1 Antibody Pembrolizumab. CPT Pharmacometrics Syst Pharmacol. 2017 Jan 1;6(1):11–20. doi: 10.1002/psp4.12130 27863176 PMC5270293

[3] Ouerdani A, Struemper H, Suttle A, Ouellet D, Ribba B. Preclinical Modeling of Tumor Growth and Angiogenesis Inhibition to Describe Pazopanib Clinical Effects in Renal Cell Carcinoma. CPT Pharmacometrics Syst Pharmacol. 2015 Nov 1;4(11):660. doi: 10.1002/psp4.12001 26783502 PMC4716582

[4] Stein A, Wang W, Carter AA, Chiparus O, Hollaender N, Kim H, et al. Dynamic tumor modeling of the dose-response relationship for everolimus in metastatic renal cell carcinoma using data from the phase 3 RECORD-1 trial. BMC Cancer. 2012 Jul 23;12:311. doi: 10.1186/1471-2407-12-311 22824201 PMC3495014

[5] Wang Y, Sung C, Dartois C, Ramchandani R, Booth BP, Rock E, et al. Elucidation of relationship between tumor size and survival in non-small-cell lung cancer patients can aid early decision making in clinical drug development. Clin Pharmacol Ther. 2009 Aug 1;86(2):167–74. doi: 10.1038/clpt.2009.64 19440187

[6] Ribba B, Kaloshi G, Peyre M, Ricard D, Calvez V, Tod M, et al. A tumor growth inhibition model for low-grade glioma treated with chemotherapy or radiotherapy. Clin Cancer Res. 2012 Sep 15;18(18):5071–80. doi: 10.1158/1078-0432.CCR-12-0084 22761472

[7] Ouerdani A, Goutagny S, Kalamarides M, Trocóniz IF, Ribba B. Mechanism-based modeling of the clinical effects of bevacizumab and everolimus on vestibular schwannomas of patients with neurofibromatosis type 2. Cancer Chemother Pharmacol. 2016 Jun 1;77(6):1263–73. doi: 10.1007/s00280-016-3046-2 27146400

[8] Schindler E, Amantea MA, Karlsson MO, Friberg LE. A pharmacometric framework for axitinib exposure, efficacy, and safety in metastatic renal cell carcinoma patients. CPT Pharmacometrics Syst Pharmacol. 2017 Jun 1;6(6):373–82. doi: 10.1002/psp4.12193 28378918 PMC5488123

[9] Claret L, Girard P, Hoff PM, Van Cutsem E, Zuideveld KP, Jorga K, et al. Model-based prediction of phase III overall survival in colorectal cancer on the basis of phase II tumor dynamics. J Clin Oncol. 2009 Jul 27;27(25):4103–8. doi: 10.1200/JCO.2008.21.0807 19636014

[10] Mould DR. Developing Models of Disease Progression. Pharmacometrics Sci Quant Pharmacol. 2006 May 18;547–81.

[11] Benzekry S, Lamont C, Beheshti A, Tracz A, Ebos JMLL, Hlatky L, et al. Classical Mathematical Models for Description and Prediction of Experimental Tumor Growth. PLoS Comput Biol. 2014 Aug 28;10(8):e1003800. doi: 10.1371/journal.pcbi.1003800 25167199 PMC4148196

[12] Voulgarelis D, Bulusu KC, Yates JWT. Comparison of classical tumour growth models for patient derived and cell-line derived xenografts using the nonlinear mixed-effects framework. J Biol Dyn. 2022;16(1):160–85. doi: 10.1080/17513758.2022.2061615 35404766

[13] Vaghi C, Rodallec A, Fanciullino R, Ciccolini J, Mochel JP, Mastri M, et al. Population modeling of tumor growth curves and the reduced Gompertz model improve prediction of the age of experimental tumors Author summary. PLoS Comput Biol. 2020;16(2):e1007178. 32097421 10.1371/journal.pcbi.1007178 PMC7059968

[14] Garcia-Cremades M, Pitou C, Iversen PW, Troconiz IF. Characterizing gemcitabine effects administered as single agent or combined with carboplatin in mice pancreatic and ovarian cancer xenografts: A semimechanistic pharmacokinetic/pharmacodynamics tumor growth-response model. J Pharmacol Exp Ther. 2017 Mar 1;360(3):445–56. doi: 10.1124/jpet.116.237610 28028124

[15] Hutchinson LG, Mueller HJ, Gaffney EA, Maini PK, Wagg J, Phipps A, et al. Modeling longitudinal preclinical tumor size data to identify transient dynamics in tumor response to Antiangiogenic Drugs. CPT Pharmacometrics Syst Pharmacol. 2016 Nov 1;5(11):636–45. doi: 10.1002/psp4.12142 27863175 PMC5192995

[16] Whiteside TL. The tumor microenvironment and its role in promoting tumor growth. Vol. 27, Oncogene. Nature Publishing Group; 2008. p. 5904–12. 18836471 10.1038/onc.2008.271 PMC3689267

[17] Hinshaw DC, Shevde LA. The tumor microenvironment innately modulates cancer progression. Vol. 79, Cancer Research. American Association for Cancer Research Inc.; 2019. p. 4557–67. 10.1158/0008-5472.CAN-18-3962 PMC6744958 31350295

[18] Emami Nejad A, Najafgholian S, Rostami A, Sistani A, Shojaeifar S, Esparvarinha M, et al. The role of hypoxia in the tumor microenvironment and development of cancer stem cell: a novel approach to developing treatment. Cancer Cell Int 2021 211. 2021 Jan 20;21(1):1–26. doi: 10.1186/s12935-020-01719-5 33472628 PMC7816485

[19] Yotnda P, Wu D, Swanson AM. Hypoxic tumors and their effect on immune cells and cancer therapy. Vol. 651, Methods in molecular biology (Clifton N.J.). Humana Press, Totowa, NJ; 2010. p. 1–29. 20686957 10.1007/978-1-60761-786-0_1

[20] Kareva I, Berezovskaya F. Cancer immunoediting: A process driven by metabolic competition as a predator–prey–shared resource type model. J Theor Biol. 2015 Sep 7;380:463–72. doi: 10.1016/j.jtbi.2015.06.007 26116366

[21] Kareva I, Luddy KA, O’Farrelly C, Gatenby RA, Brown JS. Predator-Prey in Tumor-Immune Interactions: A Wrong Model or Just an Incomplete One? Front Immunol. 2021 Aug 31;12:3391. doi: 10.3389/fimmu.2021.668221 34531851 PMC8438324

[22] Hamilton PT, Anholt BR, Nelson BH. Tumour immunotherapy: lessons from predator–prey theory. Nat Rev Immunol 2022. 2022 May 5;1–11. doi: 10.1038/s41577-022-00719-y 35513493

[23] Parra-Guillen ZP, Mangas-Sanjuan V, Garcia-Cremades M, Troconiz IF, Mo G, Pitou C, et al. Systematic modeling and design evaluation of unperturbed tumor dynamics in xenografts. J Pharmacol Exp Ther. 2018;366(1):96–104. doi: 10.1124/jpet.118.248286 29691287

[24] Pierrillas PB, Tod M, Amiel M, Chenel M, Henin E. Improvement of Parameter Estimations in Tumor Growth Inhibition Models on Xenografted Animals: Handling Sacrifice Censoring and Error Caused by Experimental Measurement on Larger Tumor Sizes. AAPS J. 2016 Sep 1;18(5):1262–72. doi: 10.1208/s12248-016-9936-8 27329303

[25] 1994–2022 The MathWorks I. Cubic spline data interpolation—MATLAB [Internet]. Vol. 1. 2012 [cited 2022 Dec 18]. p. 1–6. Available from: https://es.mathworks.com/help/curvefit/spaps.html.

[26] Lindstrom MJ, Bates DM. Nonlinear Mixed Effects Models for Repeated Measures Data. Biometrics. 1990 Sep;46(3):673. 2242409

[27] Antony, France: Lixoft SAS 2021. Monolix version 2021R1. Antony, France: Lixoft SAS, 2021; 2021.

[28] Mack CA. CHE384, From Data to Decisions: Measurement, Uncertainty, Analysis, and Modeling. Independence of Residuals. Texas, Austin: The University of Texas; 2016.

[29] Sobol IM. Global sensitivity indices for nonlinear mathematical models and their Monte Carlo estimates. Math Comput Simul. 2001 Feb 15;55(1–3):271–80.

[30] Saltelli A, Ratto M, Andres T, Campolongo F, Cariboni J, Gatelli D, et al. Global Sensitivity Analysis. The Primer. Glob Sensit Anal Prim. 2007;183–236.

[31] Pianosi F, Sarrazin F, Wagener T. A Matlab toolbox for Global Sensitivity Analysis. Environ Model Softw. 2015 Aug 1;70:80–5.

[32] Simeoni M, Magni P, Cammia C, De Nicolao G, Croci V, Pesenti E, et al. Predictive Pharmacokinetic-Pharmacodynamic Modeling of Tumor Growth Kinetics in Xenograft Models after Administration of Anticancer Agents. Cancer Res. 2004 Feb 1;64(3):1094–101. doi: 10.1158/0008-5472.can-03-2524 14871843

[33] de Pillis LG, Gu W, Fister KR, Head T, Maples K, Murugan A, et al. Chemotherapy for tumors: An analysis of the dynamics and a study of quadratic and linear optimal controls. Math Biosci. 2007 Sep;209(1):292–315. doi: 10.1016/j.mbs.2006.05.003 17306310

[34] De Pillis LG, Radunskaya A. A mathematical tumor model with immune resistance and drug therapy: An optimal control approach. J Theor Med. 2001;3(2):79–100.

[35] Panetta JC, Schaiquevich P, Santana VM, Stewart CF. Using pharmacokinetic and pharmacodynamic modeling and simulation to evaluate importance of schedule in topotecan therapy for pediatric neuroblastoma. Clin Cancer Res. 2008 Jan 1;14(1):318–25. doi: 10.1158/1078-0432.CCR-07-1243 18172284

[36] Hahnfeldt P, Panigrahy D, Folkman J, Hlatky L. Tumor development under angiogenic signaling: A dynamical theory of tumor growth, treatment response, and postvascular dormancy. Cancer Res. 1999;59(19):4770–5. 10519381

[37] Falzone L, Salomone S, Libra M. Evolution of cancer pharmacological treatments at the turn of the third millennium. Front Pharmacol. 2018 Nov 13;9(NOV):1300. doi: 10.3389/fphar.2018.01300 30483135 PMC6243123

[38] Imbs DC, Cheikh R El, Boyer A, Ciccolini J, Mascaux C, Lacarelle B, et al. Revisiting Bevacizumab + Cytotoxics Scheduling Using Mathematical Modeling: Proof of Concept Study in Experimental Non-Small Cell Lung Carcinoma. CPT Pharmacometrics Syst Pharmacol. 2018 Jan 1;7(1):42. 29218795 10.1002/psp4.12265 PMC5784740

[39] Norton L. A Gompertzian Model of Human Breast Cancer Growth. Cancer Res. 1988;48:7067–71. 3191483

[40] Lestini G, Mentré F, Magni P. Optimal Design for Informative Protocols in Xenograft Tumor Growth Inhibition Experiments in Mice. 10.1208/s12248-016-9924-z PMC5660732 27306546

[41] Stamper IJ, Owen MR, Maini PK, Byrne HM. Oscillatory dynamics in a model of vascular tumour growth—implications for chemotherapy. Biol Direct. 2010 Apr 20;5(1):1–17. 20406447 10.1186/1745-6150-5-27 PMC2877015

[42] Vilanova G, Colominas I, Gomez H. A mathematical model of tumour angiogenesis: Growth, regression and regrowth. J R Soc Interface. 2017 Jan 1;14(126). doi: 10.1098/rsif.2016.0918 28100829 PMC5310739

[43] Kirschner D, Panetta JC. Modeling immunotherapy of the tumor—Immune interaction. J Math Biol. 1998;37(3):235–52. doi: 10.1007/s002850050127 9785481

[44] Robertson-Tessi M, El-Kareh A, Goriely A. A mathematical model of tumor-immune interactions. J Theor Biol. 2012;294:56–73. doi: 10.1016/j.jtbi.2011.10.027 22051568

[45] Mangas-Sanjuan V, Buil-Bruna N, Garrido MJ, Soto E, Trocóniz IF. Semimechanistic cell-cycle type-based pharmacokinetic/pharmacodynamic model of chemotherapy-induced neutropenic effects of diflomotecan under different dosing schedules. J Pharmacol Exp Ther. 2015 Jul 1;354(1):55–64. doi: 10.1124/jpet.115.223776 25948593

