# Supplementary material for: Mechanistic characterization of oscillatory patterns in unperturbed tumor growth dynamics: The interplay between cancer cells and components of tumor microenvironment
Source: PLoS Comput Biol. 2023 Oct 4;19(10):e1011507. doi: 10.1371/journal.pcbi.1011507 (PMC10550146; doi:10.1371/journal.pcbi.1011507)
Supplement: S1 Text — (DOCX) [file pcbi.1011507.s001.docx]

**S1 Text – RAW TUMOR VOLUME DATA AND EMPIRICAL EXPLORATORY ANALYSIS OF TUMOR VOLUME PROFILES**

The experimental data presented in this work has been previously published by Parra et al.(1). Overall, it includes data from 10 different tumor types and 12 different cell lines (see Table A for additional details regarding number of animals and samples in each experiment).

**Table A**. Summary of the experimental data available for each of the tumor cell lines.

| Tumor | Cell line | Number of mice | Number of samples |
| --- | --- | --- | --- |
| Breast | MB-231 | 6 | 48 |
| Leukemia | MV411 | 8 | 78 |
| Lung | A549 | 6 | 40 |
|  | Calu-6 | 5 | 35 |
|  | H1650 | 10 | 130 |
|  | H1975 | 3 | 27 |
|  | H2122 | 4 | 28 |
|  | H441 | 12 | 106 |
| Lymphoma | JEKO-1 | 7 | 78 |
| Melanoma | A2058 | 2 | 18 |
|  | A375 | 4 | 51 |
| Pancreas | MIA PaCa-2 | 3 | 24 |

The computation of the half-periods (HP) for all the 85 mice is summarized in the boxplots of S1 Fig. We recall that the HP were obtained from the analysis of the residual curves (see Fig 4A right panel, in the main text) for each individual. In S1 Fig, the collected information of the HP is represented for all the mice according to the two selected points: extrema (i.e., HP calculated as time interval between two consecutive local extrema (maximum and minimum) (left panel), and zero-crossing (i.e., HP obtained as time interval between two consecutive crossings with the zero-line) (right panel). These points were computed for both log transformed data (left boxplots of each panel) and unit normalization data (right boxplots of each panel). Table B provides statistical summaries of the data shown in S1 Fig.

**Table B.** Statistical summary of the estimated half-periods (HPs) according to the four different techniques described in the text: two data transformation (unit normalization and log) and two different points (zero-crossing and extrema).

| **Data transformation** | **Points** | **Mean** | **Standard deviation** |
| --- | --- | --- | --- |
| Unit normalization | Zero-crossing | 10.5 | 4.72 |
|  | Extrema | 8.00 | 3.95 |
| Log transformation | Zero-crossing | 10.9 | 5.32 |
|  | Extrema | 8.25 | 4.47 |

In the next step of the exploratory data analysis we tested whether oscillations were random or on the contrary they were due to an underlying mechanism. For this purpose, as it has been mentioned in the main text, we used Monte-Carlo simulations. More concretely, for each individual, we perturbed the classical best fit with a Gaussian white noise at each data point with a variance equal to the variance measured in the data. Subsequently, we performed

5,000 repetitions of the data + noise perturbation for each individual. analyzed each dataset using the same algorithm as for the original data (in the exploratory first phase). Afterwards, half-period were computed and used to compare the distribution (histogram) of these simulated HP with the original HP. We observed that the measured original HP lie mostly in the extreme tail of the simulated distribution. This confirms that indeed the original HP were not caused by noise alone.

S2 Fig shows the distribution of HP resulting from random simulations performed with noise computed after the unit normalization transformation for both points: zero-crossing (left panel), and extrema (right panel) for a single mouse. It is also noticeable that the HP from the original data (colored symbols, red cross or green stars) of the case example of Fig 3 (main text), are clearly in the right tail of the simulated distribution of HP. Concretely, we obtained three HP computed with the zero-crossing (11.8 days; 12.4 days; 14.2 days), and two HP with the extrema (11.4 days; 16.2 days). In addition, the numerical values indicated in each panel correspond to the proportion of simulated data that are above the corresponding original HP. The fact that observed values are in the right tail of the simulated distribution supports the hypothesis that noise is not the only cause of the detected oscillations. Lastly, we rigorously confirmed this fact using a Kolmogorov-Smirnov hypothesis test.

In the next step of the exploratory analysis, we collected the estimated proportions calculated from the simulated data of the 85 individuals (Fig 4C in the main text), and we tested using Kolmogorov-Smirnov (KS), the following hypothesis: H_0_ (null hypothesis) = the noise is the sole responsible for the observed oscillations. This implies that under the null hypothesis, the estimated proportions are distributed uniformly. The computed KS p-values are lower than 10^-9^ in both situations. Therefore, the null-hypothesis, which stated that the oscillations are due to noise, can be safely rejected.

**REFERENCES**

1. Parra-Guillen ZP, Mangas-Sanjuan V, Garcia-Cremades M, Troconiz IF, Mo G, Pitou C, et al. Systematic modeling and design evaluation of unperturbed tumor dynamics in xenografts. J Pharmacol Exp Ther. 2018;366:96–104.
