# Supplementary material for: Mechanistic characterization of oscillatory patterns in unperturbed tumor growth dynamics: The interplay between cancer cells and components of tumor microenvironment
Source: PLoS Comput Biol. 2023 Oct 4;19(10):e1011507. doi: 10.1371/journal.pcbi.1011507 (PMC10550146; doi:10.1371/journal.pcbi.1011507)
Supplement: S2 Text — (DOCX) [file pcbi.1011507.s002.docx]

**S2 Text – TUMOR GROWTH DYNAMIC MODELS**

**A. CLASSICAL TUMOR GROWTH MODELS**

In this work, several classical tumor growth models including the linear, exponential, Gompertz and Simeoni equations, were fitted to our data. Table A includes the model equations and a brief model description, the AIC values and final parameters for the explored models.

**Table A.** Summary of model development based on AIC values.

| TG Model | | Linear | Exponential | Gompertz | Simeoni |
| --- | --- | --- | --- | --- | --- |
| Model description | | | | | |
|  | | Tumor growth independent of TV (1,2) | The tumor grows proportionally to its volume at a constant rate (3,4) | Progressive decrease of the growth rate as the tumor gets larger. TV converges to a carrying capacity (5,6) | An initial exponential increase in TV followed by linear growth (7,8) |
| Equations ($\frac{dTV}{dt}=$) | | | | | |
|  | | $kgl$ | $kge\times TV$ | $kge\times TV \times\log\left( \frac{{TV}_{max}}{TV} \right)$ | $\frac{kge\times TV}{\left\{ 1+ \left[ \left( \frac{kge}{kgl} \right)\times TV \right]^{20} \right\}^{\frac{1}{20}}}$ |
| AIC | |  |  |  |  |
|  | | 1606.5 | 563.96 | 403.59 | 194.45 |
| Parameters | |  |  |  |  |
|  | Estimates (RSE%) | | | | |
| TV0 (mm^3^) | | 1 Fix | 69.55 (5) | 47.2 (13) | 50.12 (10) |
| kgl  (mm^3^×day^-1^) | | 16.39 (6) |  |  | 46.67 (30) |
| Kge (day^-1^) | |  | 0.06 (4) | 0.015 (7) | 0.085 (47) |
| TVmax (mm^3^) | |  |  | 35153.95 (27) |  |
|  | Inter-animal variability (RSE%) | | | | |
| IAV TV0 | | NE | 0.42 (9) | 0.5 (9) | 0.4 (10) |
| IAV kgl | | 0.52 (8) |  |  | 1.12 (9) |
| IAV kge | |  | 0.36 (8) | 0.46 (11) | 0.35 (9) |
|  | Residual error | | | | |
| a (log(mm^3^)) | | 0.46 (2) | 0.24 (2) | 0.22 (2) | 0.19 (2) |
| Typical profile^1^ | |  |  |  |  |
|  | | 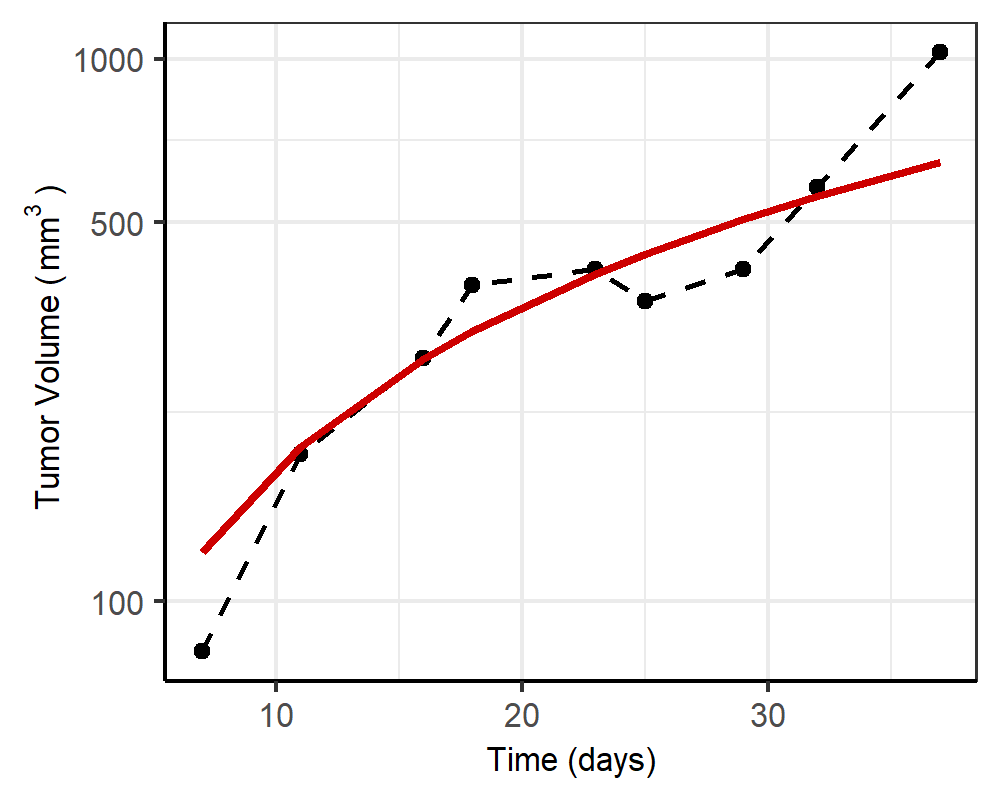 | 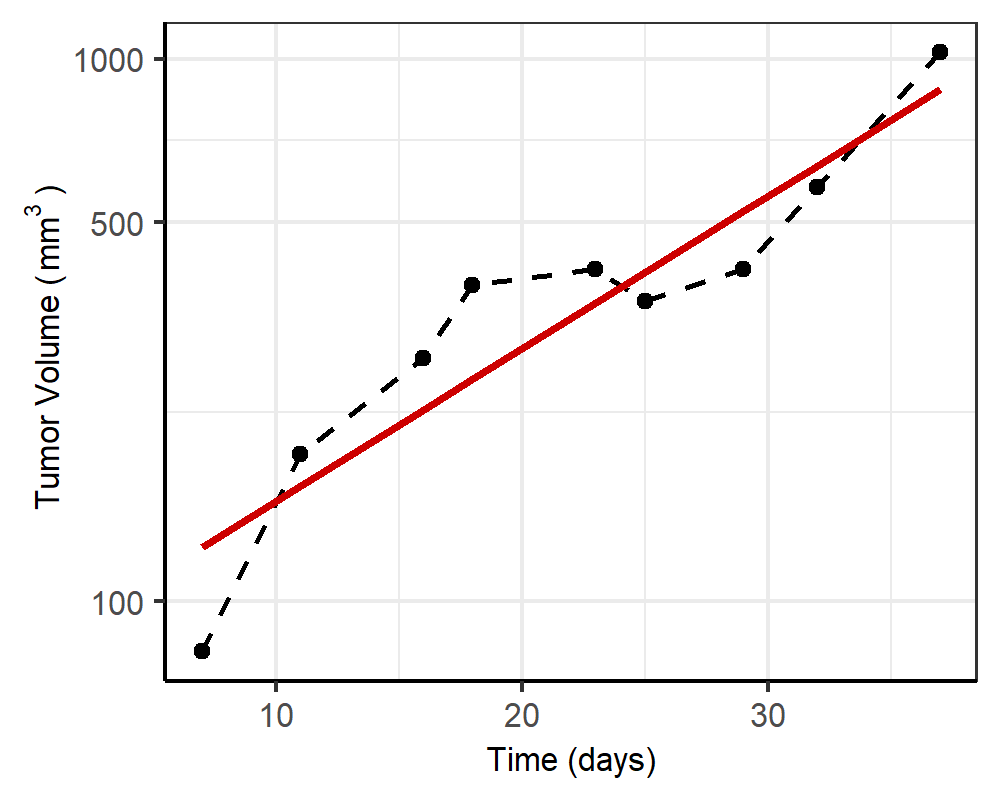 | 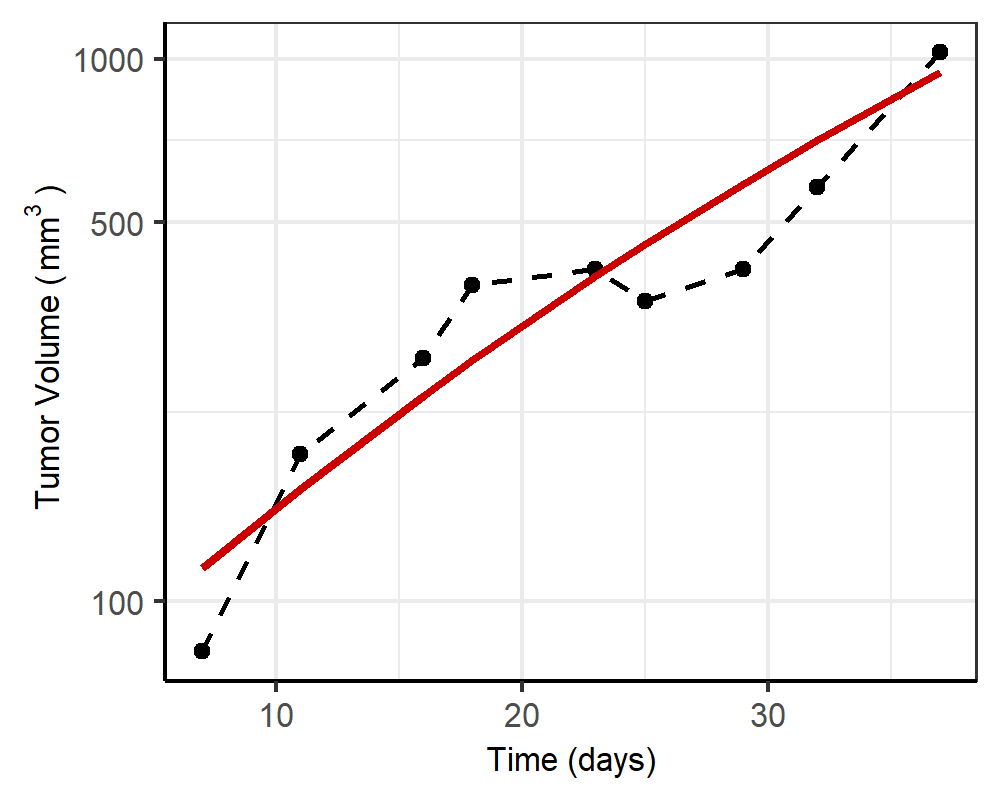 | 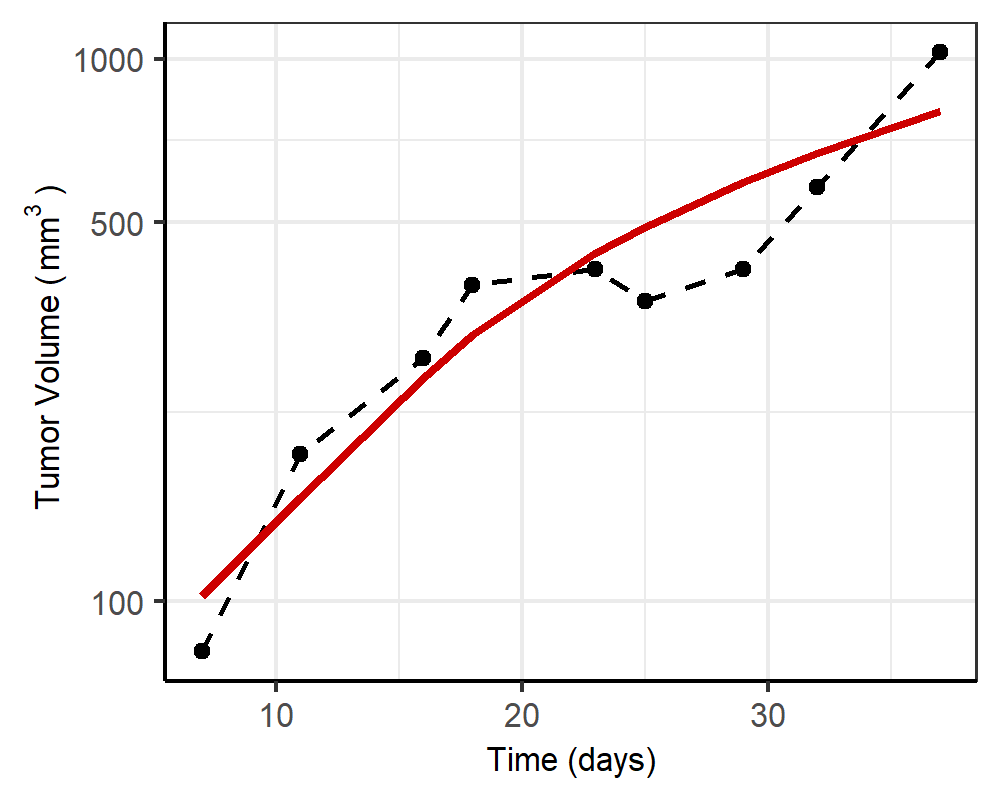 |
| TV, tumor volume at any time after cell inoculation; kgl, linear growth rate; kge, exponential growth rate constant; TV_max_, carrying capacity  ^1^The full dataset was fitted to each model presented in the equation. The dots represent the tumor volume observation for a single mouse, the dashed line reflects the observed tumor volume profile and the red line represent the tumor volume profile of a mice with the population parameter estimates. | | | | | |

**B. TUMOR GROWTH MODELS INCLUDING BIOLOGICAL MECHANISMS**

During model development, different structures integrating biologic processes such as the angiogenesis, the immune system or the heterogeneity among tumor cells, were also investigated and fitted to the data. Table B details the three models included in our analysis. However, the non-monotonic behavior was not captured by none of them (see the typical profile of TV for each model together with a selected individual in Table B).

**Table B.** Description of the different monotonic tumor growth models used in this analysis.

| Model | Equation | Model description | Typical profile^1^ | Ref. |
| --- | --- | --- | --- | --- |
| Tumor growth models integrating biological processes | | | | |
| Angiogenesis | $\frac{dTV}{dt}=\left[ growth \right]$  $\frac{dK}{dt}=-\lambda_{2}\times K+b\times TV-d\times K\times{TV}^{2/3}$ | The carrying capacity (TV_max_) of the tumor is determined by the tumor vasculature that is in turn affected by the TV | 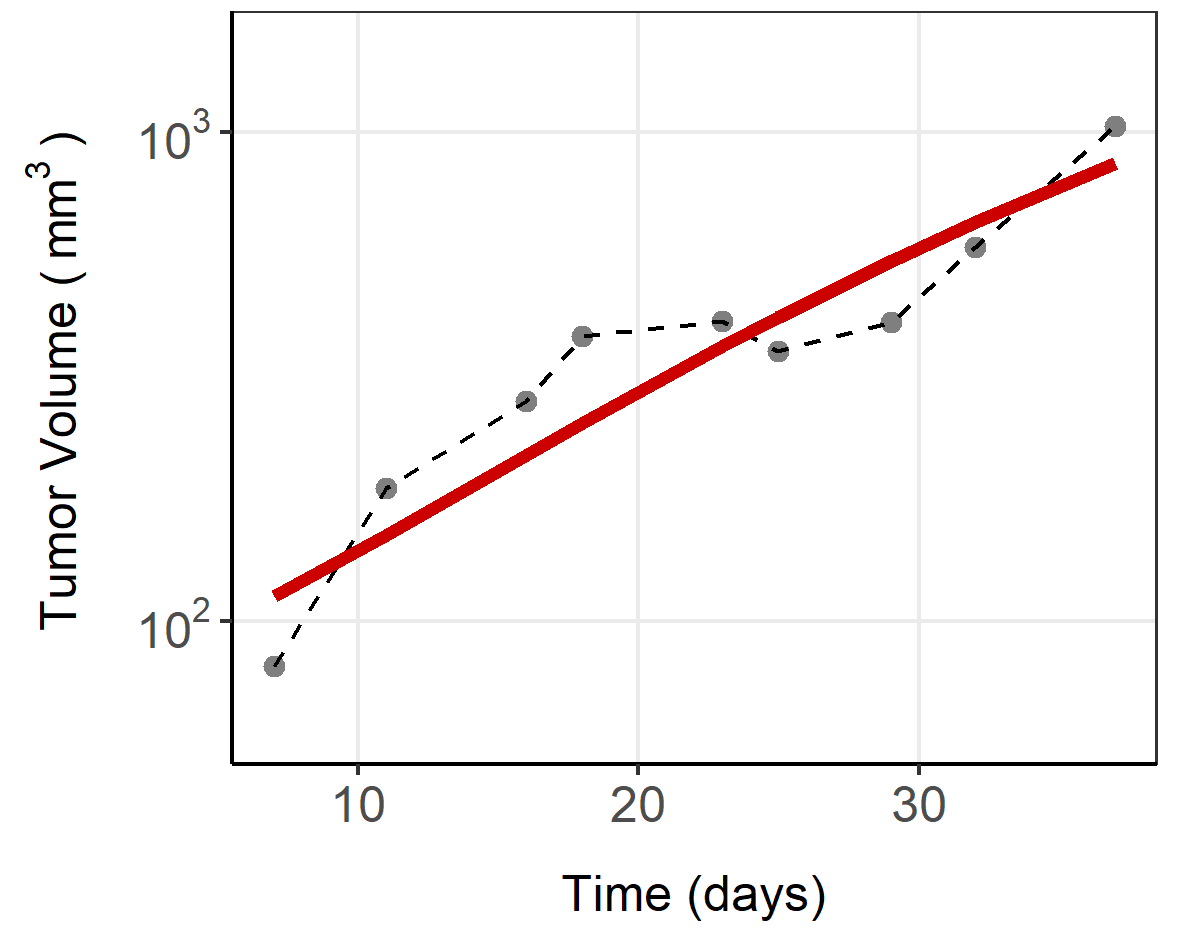 | (9,10) |
| Immune system | $\frac{dTV}{dt}=\left[ growth \right]-ki\times I\times TV$  $\frac{dI}{dt}={kp}_{i}-{kd}_{i}\times I+g\times\frac{TV}{h+TV}\times I-p \times I\times TV$ | Tumor growth is assumed to be controlled by the immune system. Additionally, immune cells are recruited by the tumor through a Michaelis-Mentel term and inactivated through contact with tumor cells. | 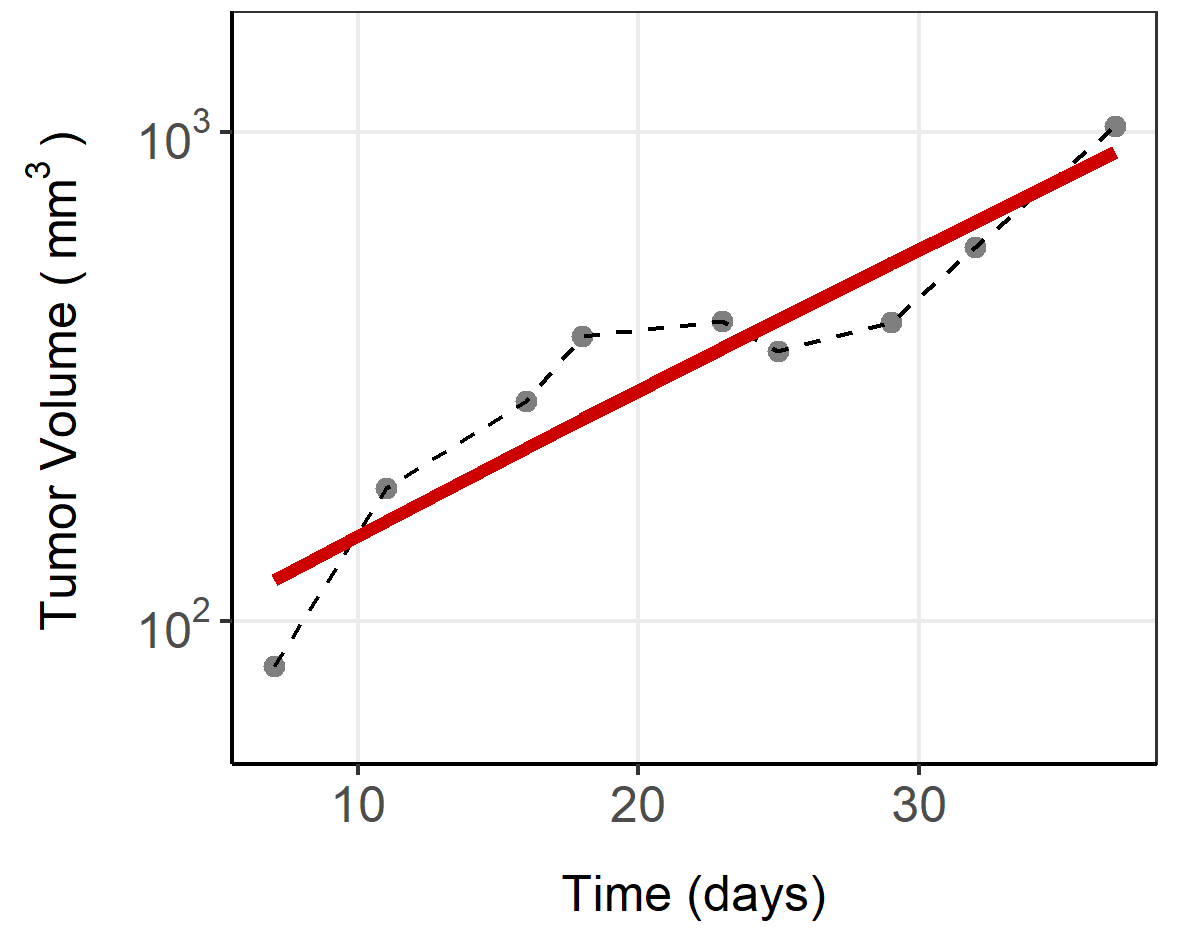 | (11,12) |
| Heterogeneity | $\frac{dP}{dt}=\left[ growth \right]-k_{pq}\times Q$  $\frac{dQ}{dt}= k_{pq}\times Q$  $TV= Q+P$ | Quiescent (Q) tumor cells are assumed to result from a first-order conversion from proliferative (P) tumor cells. | 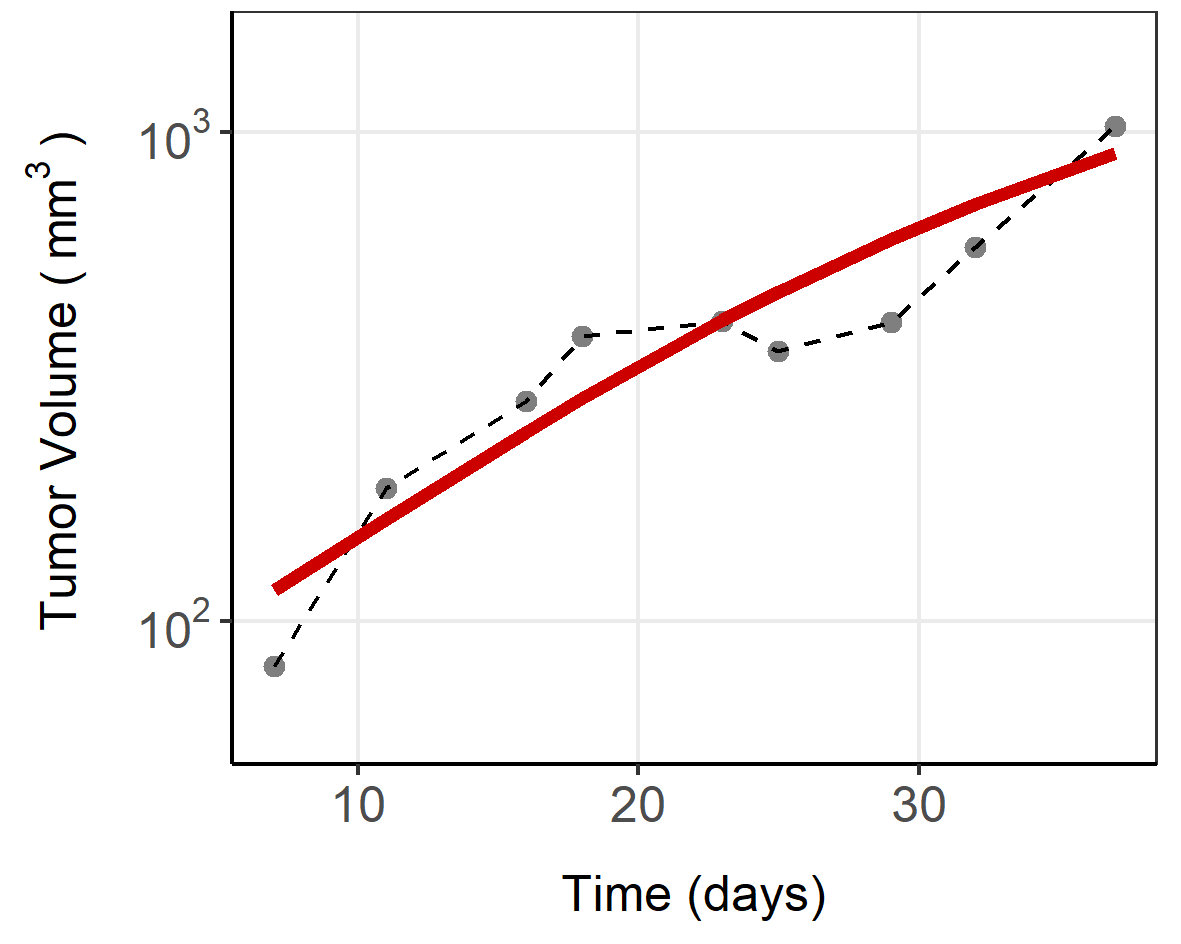 | (4,13) |
| TV, tumor volume at any time after cell inoculation; kgl, linear growth rate; kge, exponential growth rate constant; TV_max_, carrying capacity; kpq, transit constant between two compartments; ki, degradation constant by immune cell: kpi, proliferative immune cells constant; kdi, immune cells death constant; h and g, Michaelis-Mentel terms of immune cells recruitment by the tumor; p, inactivation rate constant of immune cells by the tumor  ^1^The full dataset was fitted to each model presented in the equation. The dots represent the tumor volume observation for a single mouse, the dashed line reflects the observed tumor volume profile and the red line represent the tumor volume profile of a mice with the population parameter estimates. | | | | |

**C. ANALYSIS OF THE TUMOR GROWTH MODEL**

The dynamics observed in the time evolution of the raw data is based on the three-dimensional system of ordinary differential equations (ODEs). The final estimated model parameters can be found in Table 1 (main text). Additionally, in order to further understand the dynamics of the system and the interplay between the tumor, the resources and the angiogenesis, a brief mathematical analysis was performed.

In the following section, we are going to describe the equilibrium points and the local stability of the system of ODEs:

$$\frac{dTV}{dt}=\lambda\times TV\times RES -k_{death} \times TV \left( 1 \right)$$

$$\frac{dANG}{dt} =k_{ang}\times TV- k_{death}\times ANG (2)$$

$$\frac{dRES}{dt} =k_{res}\times ANG- k_{consumption}\times TV (3)$$

The equilibrium points are obtained by solving the following system:

$$\left\{ \begin{matrix} 0 & =\lambda\times TV\times RES -k_{death} \times TV \\ 0 & =k_{ang}\times TV- k_{death}\times ANG \\ 0 & = k_{res}\times ANG- k_{consumption}\times TV \end{matrix} (4) \right.$$

The two equilibrium points are, $TV\text{=} ANG=0 ; RES={RES}_{0}$ (trivial solution); and a degenerated solution provided that the coefficients satisfy the following condition:

$$\frac{k_{death}}{k_{ang}}=\frac{k_{res}}{k_{consumption}}$$

that is given by :

$$\left\{ \begin{aligned} ANG\mathbb{\in R} \\ TV=\frac{k_{death} ANG}{k_{ang}} \\ RES=\frac{k_{death}}{\lambda} \end{aligned} \right.$$

The local equilibrium can be assessed through the computation of the eigenvalues of the corresponding matrix:

The Jacobian matrix for the trivial solution is given by:

$$J_{0}=\left[ \begin{matrix} {\lambda{RES}_{0}-k}_{death} & 0 & 0 \\ k_{ang} & {-k}_{death} & 0 \\ -k_{consum.} & k_{res} & 0 \end{matrix} \right]$$

Since the matrix is triangular, the three eigenvalues are given by the diagonal elements of the matrix:

$$0; {-k}_{death}; {\lambda{RES}_{0}-k}_{death}$$

The stability of the trivial equilibrium point is thus ensured if

$${\lambda{RES}_{0}-k}_{death}<0$$

For the degenerate solution, the Jacobian matrix is given by:

$$J_{1}=\left[ \begin{matrix} 0 & 0 & \frac{{\lambda k}_{death} ANG}{k_{ang}} \\ k_{ang} & {-k}_{death} & 0 \\ -k_{consum.} & k_{res} & 0 \end{matrix} \right]$$

The eigenvalues of the matrix are given:

$$0; \frac{{-k}_{death}\pm\sqrt{\Delta}}{2}$$

where the discriminant Δ is given by:

$$\Delta=k_{death}^{2}-4 \lambda k_{res}ANG$$

Thus, this degenerate solution presents oscillations whenever we have complex eigenvalues which happens when:

$$ANG> \frac{k_{death}^{2}}{4 \lambda k_{res}}$$

The estimation of the coefficients given by Monolix are collected in Table 1 and they show that we are not close to the degenerate case. However, simulations shown in Fig 6 (main text), and also S4 Fig shows that the dynamics present non-uniform oscillations (non-constant periods and amplitudes) in a neighborhood of this set of parameters.

**References**

1. Stein A, Wang W, Carter AA, Chiparus O, Hollaender N, Kim H, et al. Dynamic tumor modeling of the dose-response relationship for everolimus in metastatic renal cell carcinoma using data from the phase 3 RECORD-1 trial. BMC Cancer. BioMed Central; 2012;12:311.

2. De Buck SS, Jakab A, Boehm M, Bootle D, Juric D, Quadt C, et al. Population pharmacokinetics and pharmacodynamics of BYL719, a phosphoinositide 3-kinase antagonist, in adult patients with advanced solid malignancies. Br J Clin Pharmacol. Wiley-Blackwell; 2014;78:543–55.

3. Claret L, Girard P, Hoff PM, Van Cutsem E, Zuideveld KP, Jorga K, et al. Model-based prediction of phase III overall survival in colorectal cancer on the basis of phase II tumor dynamics. J Clin Oncol. American Society of Clinical Oncology; 2009;27:4103–8.

4. Panetta JC, Schaiquevich P, Santana VM, Stewart CF. Using pharmacokinetic and pharmacodynamic modeling and simulation to evaluate importance of schedule in topotecan therapy for pediatric neuroblastoma. Clin Cancer Res. American Association for Cancer Research; 2008;14:318–25.

5. Tham LS, Wang L, Soo RA, Lee SC, Lee HS, Yong WP, et al. A pharmacodynamic model for the time course of tumor shrinkage by gemcitabine + carboplatin in non-small cell lung cancer patients. Clin Cancer Res. American Association for Cancer Research; 2008;14:4213–8.

6. Schneider BK, Boyer A, Ciccolini J, Barlesi F, Wang K, Benzekry S, et al. Optimal Scheduling of Bevacizumab and Pemetrexed/Cisplatin Dosing in Non‐Small Cell Lung Cancer. CPT Pharmacometrics Syst Pharmacol. Wiley-Blackwell; 2019;8:577.

7. Ouerdani A, Goutagny S, Kalamarides M, Trocóniz IF, Ribba B. Mechanism-based modeling of the clinical effects of bevacizumab and everolimus on vestibular schwannomas of patients with neurofibromatosis type 2. Cancer Chemother Pharmacol. Springer Verlag; 2016;77:1263–73.

8. Simeoni M, Magni P, Cammia C, De Nicolao G, Croci V, Pesenti E, et al. Predictive Pharmacokinetic-Pharmacodynamic Modeling of Tumor Growth Kinetics in Xenograft Models after Administration of Anticancer Agents. Cancer Res. Cancer Res; 2004;64:1094–101.

9. Hahnfeldt P, Panigrahy D, Folkman J, Hlatky L. Tumor development under angiogenic signaling: A dynamical theory of tumor growth, treatment response, and postvascular dormancy. Cancer Res. 1999;59:4770–5.

10. Garcia-Cremades M, Pitou C, Iversen PW, Troconiz IF. Characterizing gemcitabine effects administered as single agent or combined with carboplatin in mice pancreatic and ovarian cancer xenografts: A semimechanistic pharmacokinetic/pharmacodynamics tumor growth-response model. J Pharmacol Exp Ther. American Society for Pharmacology and Experimental Therapy; 2017;360:445–56.

11. de Pillis LG, Gu W, Fister KR, Head T, Maples K, Murugan A, et al. Chemotherapy for tumors: An analysis of the dynamics and a study of quadratic and linear optimal controls. Math Biosci. 2007;209:292–315.

12. De Pillis LG, Radunskaya A. A mathematical tumor model with immune resistance and drug therapy: An optimal control approach. J Theor Med. Taylor and Francis Ltd.; 2001;3:79–100.

13. Ribba B, Kaloshi G, Peyre M, Ricard D, Calvez V, Tod M, et al. A tumor growth inhibition model for low-grade glioma treated with chemotherapy or radiotherapy. Clin Cancer Res. American Association for Cancer Research; 2012;18:5071–80.
