# Supplementary figures and images for: Mechanistic characterization of oscillatory patterns in unperturbed tumor growth dynamics: The interplay between cancer cells and components of tumor microenvironment
Source: PLoS Comput Biol. 2023 Oct 4;19(10):e1011507. doi: 10.1371/journal.pcbi.1011507 (PMC10550146; doi:10.1371/journal.pcbi.1011507)

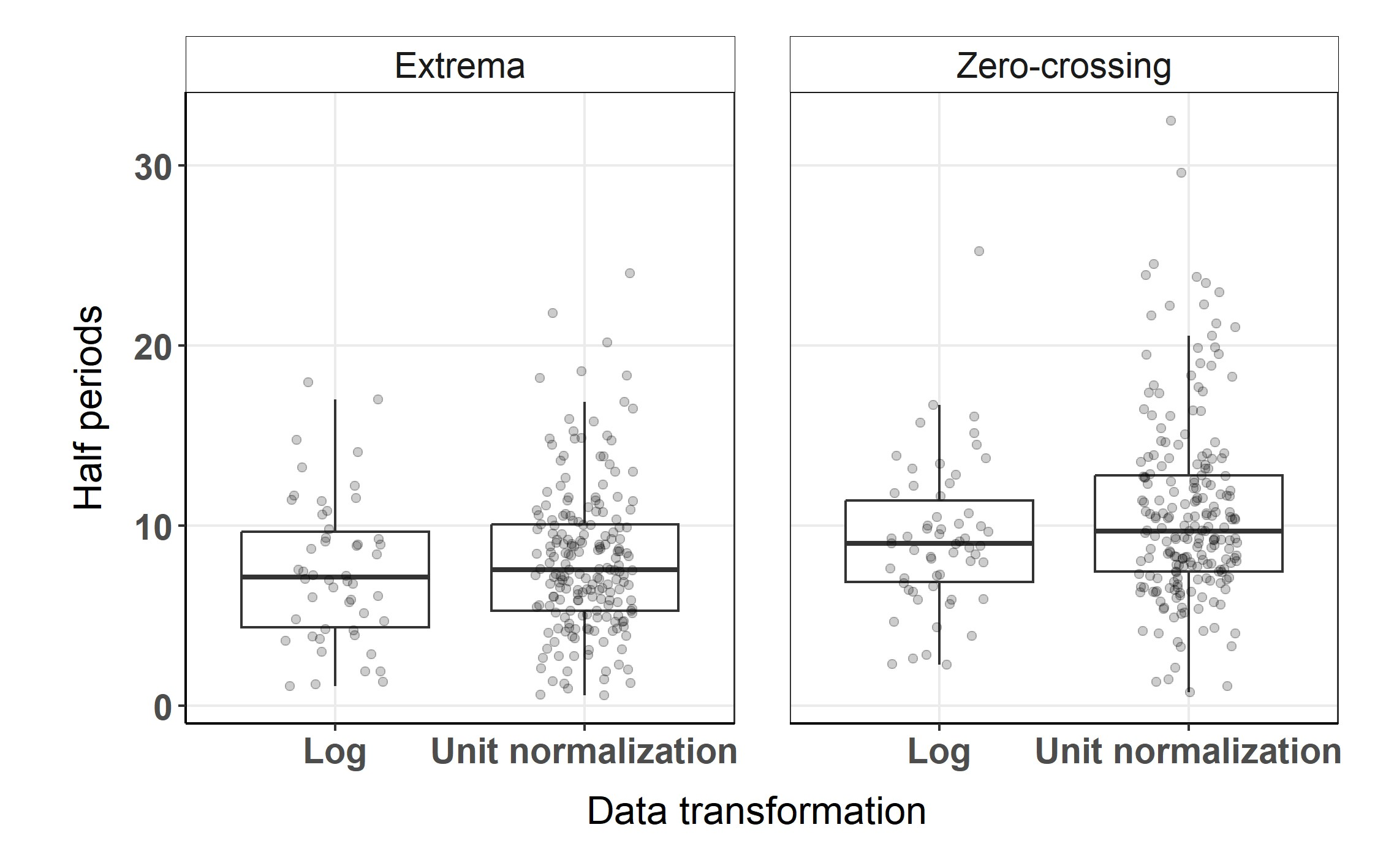

Supplement: S1 Fig — (TIF) [file pcbi.1011507.s003.tif]

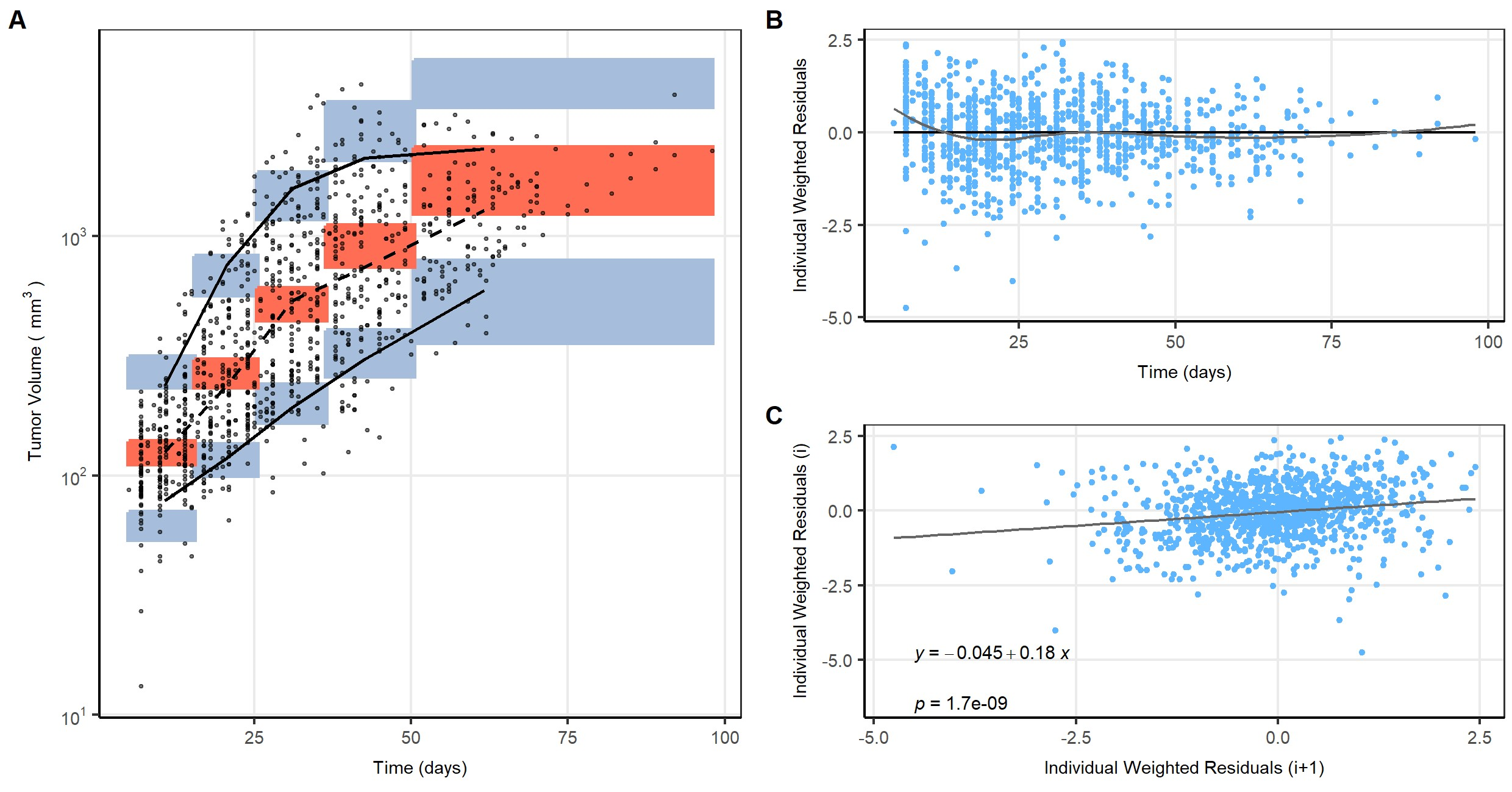

Supplement: S3 Fig — Models results and a graphical evaluation of the Simeoni tumor growth model: (A) visual predictive check: the black dots show the tumor volume measure, black lines represent the 5th, 50th and 95th percentiles of the raw data, colored areas denote the 95th confidence interval of model-predicted median (orange areas), 5th and 95th percentiles (blue areas). (B) Weighted residuals versus time. (C) Lag plot (where i represents each residual value in chronological order of observations). (TIF) [file pcbi.1011507.s005.tif]

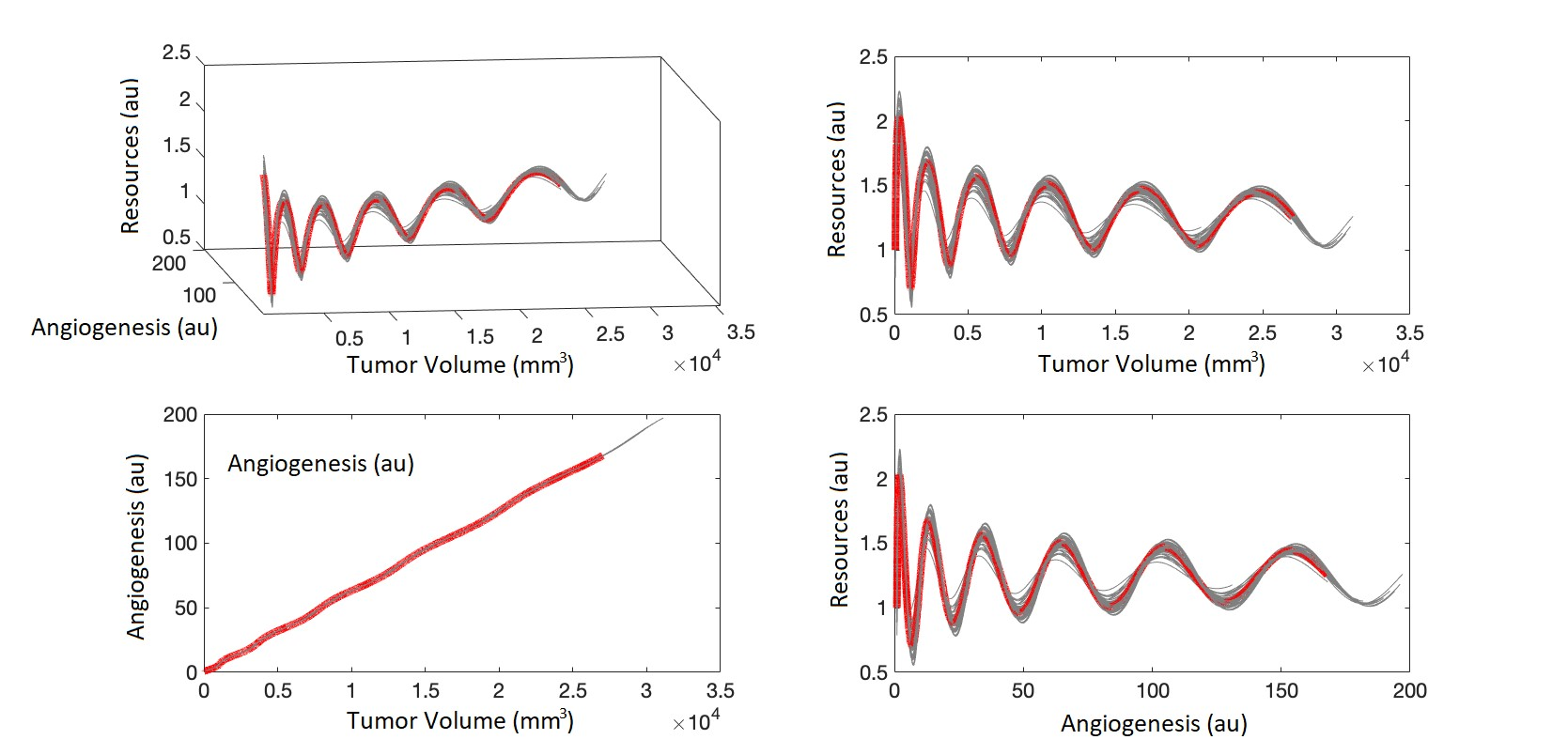

Supplement: S4 Fig — The parameters of the equations are taken from Table 1. Panel (a) shows the 3D phase space, the red curve corresponds to the main solution and the superimposed 50 thin gray lines correspond to the slightly perturbed initial conditions. Panels (b-d) correspond to the 2D projections of the solutions shown in panel (a). (TIF) [file pcbi.1011507.s006.tif]
